# Chitosan Enriched Three-Dimensional Matrix Reduces Inflammatory and Catabolic Mediators Production by Human Chondrocytes

**DOI:** 10.1371/journal.pone.0128362

**Published:** 2015-05-28

**Authors:** Frederic Oprenyeszk, Christelle Sanchez, Jean-Emile Dubuc, Véronique Maquet, Catherine Henrist, Philippe Compère, Yves Henrotin

**Affiliations:** 1 Bone and Cartilage Research Unit, Arthropôle Liege, University of Liege, Liege, Belgium; 2 Orthopaedic Department, Cliniques Universitaires St Luc, Brussels, Belgium; 3 KitoZyme SA, Parc industriel des Haut-Sarts, Herstal, Belgium; 4 Group of Research in Energy and Environment from Materials and Center for Applied Technology in Microscopy, University of Liege, Liege, Belgium; 5 Laboratory of Functional and Evolutive Morphology, Department of Environmental Sciences and Management, University of Liege, Liege, Belgium; Dasman Diabetes Institute, KUWAIT

## Abstract

This *in vitro* study investigated the metabolism of human osteoarthritic (OA) chondrocytes encapsulated in a spherical matrix enriched of chitosan. Human OA chondrocytes were encapsulated and cultured for 28 days either in chitosan-alginate beads or in alginate beads. The beads were formed by slowly passing dropwise either the chitosan 0.6%–alginate 1.2% or the alginate 1.2% solution through a syringe into a 102 mM CaCl2 solution. Beads were analyzed histologically after 28 days. Interleukin (IL)-6 and -8, prostaglandin (PG) E_2_, matrix metalloproteinases (MMPs), hyaluronan and aggrecan were quantified directly in the culture supernatant by specific ELISA and nitric oxide (NO) by using a colorimetric method based on the Griess reaction. Hematoxylin and eosin staining showed that chitosan was homogeneously distributed through the matrix and was in direct contact with chondrocytes. The production of IL-6, IL-8 and MMP-3 by chondrocytes significantly decreased in chitosan-alginate beads compared to alginate beads. PGE_2_ and NO decreased also significantly but only during the first three days of culture. Hyaluronan and aggrecan production tended to increase in chitosan-alginate beads after 28 days of culture. Chitosan-alginate beads reduced the production of inflammatory and catabolic mediators by OA chondrocytes and tended to stimulate the synthesis of cartilage matrix components. These particular effects indicate that chitosan-alginate beads are an interesting scaffold for chondrocytes encapsulation before transplantation to repair cartilage defects.

## Introduction

Osteoarthritis (OA) is a degenerative disease associated with many structural and functional disorders leading to the loss of joint integrity and function. It is the most prevalent form of arthritic disease in the world. It is mainly characterized by the slow progressive degeneration and the loss of articular cartilage accompanied by modification of the subchondral bone and synovial membrane inflammation [1]. In cartilage, an imbalance between anabolic and catabolic processes occurs and results in the progressive degradation of the extracellular matrix [2]. The catabolic response is induced by pro-inflammatory mediators (e.g. interleukin (IL)-1, IL-8, IL-6) and characterized by the overexpression of metalloproteinases (MMPs) including collagenases, stromelysin and tissue plasminogen activator, and by the production of prostaglandin (PG) E_2_ and nitric oxide (NO) [3].

Since no cure has been discovered so far for OA, the management of OA is focused on the control of symptoms, i.e. pain and loss of articular function. The guidelines recommend a combination of non-pharmacological (i.e. exercises, orthoses, insoles, weight loss) and pharmacological modalities (e.g. NSAIDS, paracetamol) as the most effective strategy to manage pain and disability associated with OA [4–10]. An alternative approach is the use of topical NSAIDs and intra-articular therapy such hyaluronic acid or glucocorticoids injection. Surgery, including arthroplasty, is recommended only as a last resort [5, 11].

Aside these treatments, debridement [12], chondral shaving, knee joint lavage can also be performed by a minimally invasive arthroscopic procedure. The purpose of these interventions is to reduce the pain and to improve the mobility. However, they are not able to restore cartilage [13, 14]. Microfracture, subchondral drilling, osteochondral allograft and autologous chondrocytes implantation assisted or not with matrix are current perspectives under investigation [15–17].

One promising approach to repair cartilage is the transplantation of autologous chondrocytes or stem cells into cartilage defect using natural or synthetic scaffold [18]. To perform this function, the scaffold must be biocompatible with the cartilaginous tissue, biodegradable, non-toxic and non-immunogenic [19]. Natural biopolymers such as alginate [20–23] and chitosan [24–27] are among the most investigated biomaterials for cartilage regeneration [28].

Chitosan is a linear natural polymer of D-glucosamine with a variable frequency of N-acetyl-D-glucosamine units that is the product obtained after chemical N-deacetylation treatment of chitin [29] which is the most abundant natural polymer after cellulose. It is present in the cell wall of fungi and the exoskeleton of shellfishes and insects. Chitosan is a fascinating candidate for biomedical applications such as biomaterials for tissue-engineered scaffold and tissue repair. Indeed, it has relevant biological proprieties such as biocompatibility, progressive degradability, absence of toxicity, lack of allergenicity and antibacterial activity [30]. Moreover, *in vitro* studies have showed that chitosan or modified chitosan promoted the expression of cartilage matrix compounds by chondrocytes [31–33]. These studies suggested that chitosan could be a good biomaterial for cartilage tissue engineering.

The aim of this work was to prepare a biocompatible three-dimensional biomaterial by mixing chitosan and alginate, and to investigate *in vitro* the biological behavior of human OA articular chondrocytes embedded in this biomaterial.

## Materials and Methods

### Specimen selection

Human cartilage was obtained from knee joints of 16 different OA patients, after total knee replacement surgery with a mean ± SEM age of 72.53 ± 7.09 and ranged between 61–83 years. Upon dissection, the femoral, tibial and patellar articular surfaces were evaluated for the pathological cartilage modifications according to the Moskowitz scale [34]. All cartilage samples showed typical OA lesions. The tissues used in this study were obtained after patient informed consent. The project was discussed with the patient verbally by the orthopedic surgeon and if the patient agreed to participate written consent was then taken. This consent procedure and the study were approved by Ethics Committee Approval of the University of Louvain (reference number B403201214793).

### Isolation of human OA chondrocytes

Cartilage was cut into small fragments and submitted to sequential enzymatic digestion with hyaluronidase, pronase and collagenase, as previously described [35]. The cells were then filtered through a nylon mesh with a pore diameter of 70 μm, and washed 2 times with 0.9% NaCl. Cell viability was estimated by trypan blue exclusion test. The chondrocytes were then pooled and used into four independent cultures (n = 4).

### Preparation of alginate and chitosan-alginate beads

The alginate beads were performed by suspending chondrocytes in 1.2% (w/v) low viscosity alginate (Sigma-Aldrich, Bornem, Belgium) solution at a density of 6 x 10^6^ cells/mL as previously described by Sanchez et al. [36].

**Fig 1:**
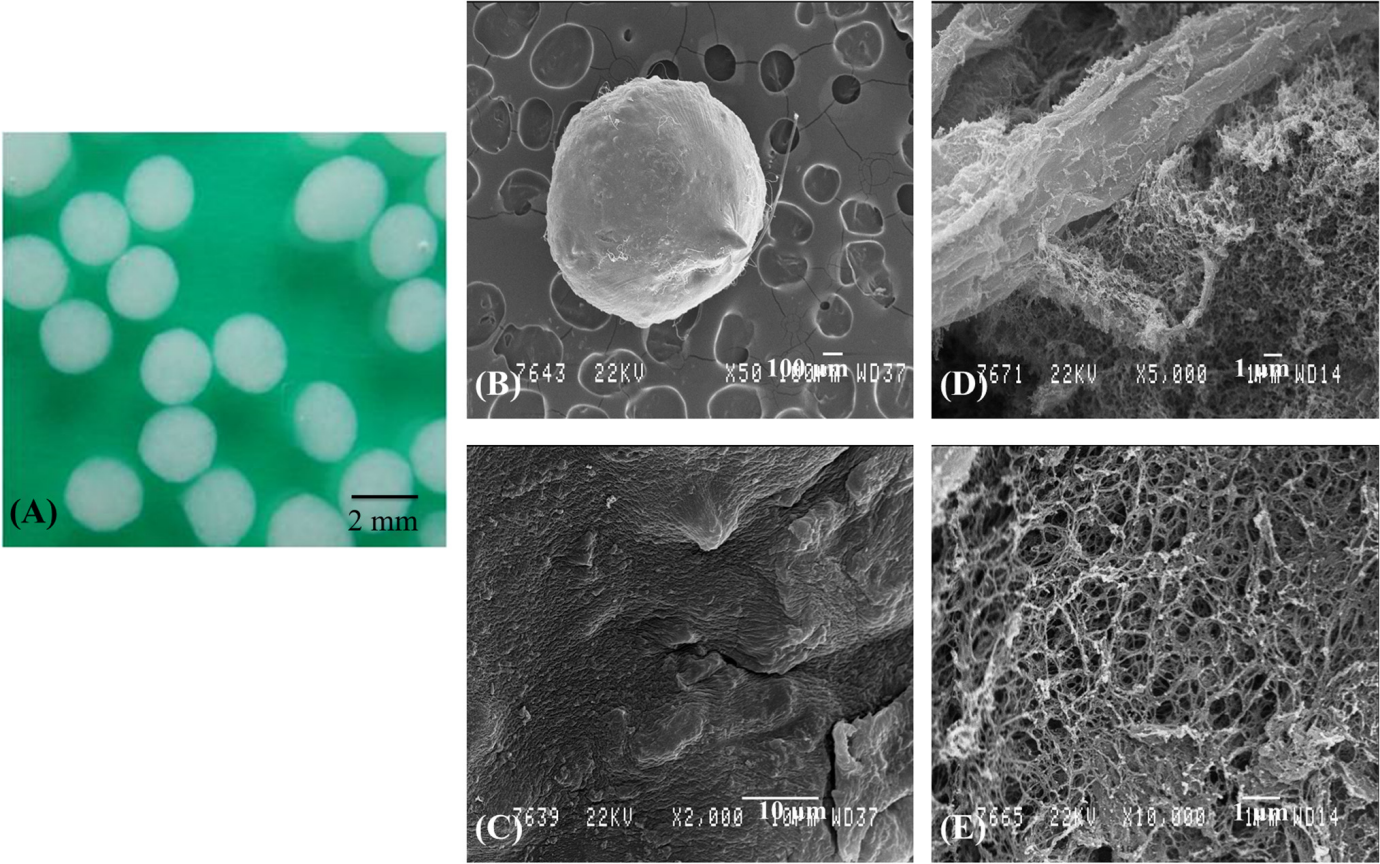
Macroscopic and electron microscopic appearance of chitosan-alginate beads. Macroscopic image of chitosan-alginate beads (A). Scanning electron micrograph of chitosan-alginate bead (B; original magnification x50) showing the surface morphology (C; original magnification x2000), the internal morphology (D; original magnification x5000) and the alginate matrix (E; original magnification x10000).

To prepare chitosan-alginate (CA) beads, 1.33% (w/v) chitosan solution and 2.4% (w/v) alginate solutions were prepared separately by dissolving 1 g of ultrapure medical-grade vegetable chitosan (KiOmedine-CsU from KitoZyme, Herstal, Belgium) in 75 mL 0.166 M acetic acid and 2 g low viscosity alginate in 83.3 mL 0.16 M NaOH. The solutions were then mixed to obtain a solution containing 0.6% (w/v) chitosan and 1.2% (w/v) alginate. Chondrocytes were suspended in the chitosan-alginate solution at the density of 6 x 10^6^ cells/mL. This mixture was slowly passed dropwise through a syringe into a 102 mM CaCl_2_ solution (Sigma). After instantaneous gelation the beads were incubated for 10 min in the CaCl_2_ solution and washed with saline solution. The chitosan used to prepare the beads had a molecular weight of 30,700 Da and 15.7 mol % of degree of acetylation. To visualize the chitosan distribution, some beads were prepared using similar chitosan labeled with fluorescein isothiocyanate (chitosan-FITC; generously provided by KitoZyme). The viscosity of chitosan-alginate solution was 294 cP (measured at 25°C with a Brookfield LVDV-II+PRO viscosimeter using a spindle 18 (Brookfield Engineering Laboratories, Middleboro, Massachusetts, USA)). The osmolarity of the chitosan-alginate solution was 256.33 ± 1.53 (mean ± SEM) mOsmol/kg using the Osmometer automatic type 15 (LÖSER; Ficsher Scientist, Aalst, Belgium). The pH, measured at 19°C with Microprocessor pH-mV Meter pH 526 (VWR, Leuven, Belgium) of chitosan-alginate solutions was 7.35 ± 0.05 (mean ± SEM). The preparation of CA beads was performed by passing droplets thought a syringe with a 25 G needle. This method produced spherical beads (Fig 1A) with a mean ± SEM diameter (measured with a digital Vernier caliper) of 2.17 ± 0.23 mm.

### Chondrocytes culture in A or CA beads

Nine beads were placed in each well of a 24 wells plate and cultured in 1 ml of DMEM (Lonza, Verviers, Belgium) supplemented with 10% foetal calf serum (Lonza), 10 mM HEPES (Lonza), 100 U/mL penicillin (Lonza), 100 μg/mL streptomycin (Lonza), 2 mM L-glutamine (Lonza), 50 μg/mL ascorbic acid (Sigma), 20 μg/mL proline (Sigma) at 37°C with 5% CO_2_ in a humidified atmosphere. The culture medium was changed twice a week and the collected supernatants were kept at -20°C until analysis. Beads were maintained in culture for up to 28 days.

Beads and supernatants were collected after key intervals throughout the culture period (i.e. 0–3, 17–21, 24–28 days). The beads were then dissolved in 1 mL 0.1 M citrate solution. The suspension was centrifuged (Centrifuge 5810, Eppendorf; VWR, Leuven, Belgium) at 300 g for 10 min. With this method, two fractions were collected: the supernatant containing macromolecules from the further-removed matrix (FRM) and a pellet containing cells with their associated cellular matrix (CM) [37]. These two fractions were studied separately. The cell pellets were washed with phosphate buffered saline (PBS; Lonza), homogenized in 1 mL Tris buffer (10 mM Tris, 50 mM KCl, pH 9) by ultrasonic dissociation at 4°C and then centrifuged at 150 g for 10 min. CM and FRM were kept at -20°C until analysis.

### Scanning electron microscopy observation

Chitosan-alginate beads were observed both in an environmental scanning electron microscope (ESEM) in low vacuum "wet" mode and in a classical scanning electron microscope (SEM) under high vacuum conditions. For environmental SEM, the wet beads were directly placed on steel stub cooled at 2°C by a "Peltier" unit in the chamber of a FEI ESEM-FEG XL-30. The observation pressure started at 7 Torr and was progressively decreased to 2 Torr leading to the slow dehydration of the sample. The images were obtained using a gaseous secondary electron detector (GSED-Electroscan) with a 500 μm pressure limiting aperture (PLA) and under 12 to 20kV accelerating voltages. For classical SEM, the chitosan-alginate beads were first dehydrated in ethanol then critical point dried in CO_2_. After mounting on an aluminum stub, they were platinum-coated (20 nm) in a Balzers SCD-030 sputtering unit. Some of them were teared to visualize their internal structure. The observations were realized in a SEM Jeol JSM-840A (Jeol, Zaventem, Belgium) under a 22kV accelerating voltage.

TEM observation was also realized on CA bead containing chondrocytes after 28 days of culture. Beads were fixed for 2 h at 4°C in 2.5% glutaraldelyde in 0.1 M sodium cacodylate (pH 7.4) and then for 1 h at 4°C in 1% osmium tetroxide in 0.1 M sodium cacodylate, pH 7.4. Samples were then dehydrated with increasing concentrations of ethanol and propylene oxide and embedded in LX112 resin (LADD, Williston, Vermont, USA) before polymerization at increasing temperatures (37°C for 24 h; 47°C for 24 h; 60°C for 48 h). For orientation purpose, 2 μm sections were performed, stained with toluidine blue, and observed by light microscopy. Ultrathin sections (50–70 nm) were cut, stained with uranyl acetate, contrasted with lead citrate, mounted on uncoated grids, and observed by TEM (FEI Tecnai G2TWIN, 200kV, Eindhoven, The Netherlands).

### Histological analysis of beads

At the end of 28 days of culture, beads were fixed with 4% paraformaldehyde in 100 mM sodium-cacodylate buffer, pH 7.4, for 4 h at 4°C; 20 mM CaCl_2_ was added to prevent disintegration of the beads. After washing overnight at 4°C in 100 mM sodium-cacodylate buffer, the beads were dehydrated in graded series of methanol, placed in a xylene wash before being embedded in paraffin. Five μm sections were cut with a microtome (Leica RM 2145; Leica, Diegem, Belgium) rehydrated and stained with hematoxylin-eosin, alcian blue or safranin-O/fast green. To visualize chitosan-FITC, hematoxylin-eosin stained sections of beads were observed using H3 filtercube (Leica). Alcian blue and safranin-O/fast green staining methods were used to analyze cartilage tissue formation. For safranin-O/fast green staining, sections were counterstained with hematoxylin (Merck, Darmstadt, Germany) and fast green (Merck) to visualize cells and cell nuclei, respectively. Safranin-O (Sigma) was used for visualization of glycosaminoglycans (GAGs) in red. Sections were also stained with alcian blue solution (1% in acetic acid) to visualize extracellular GAGs in blue.

### DNA and total protein assay

The DNA content was measured in the CM fraction using the fluorimetric method with PicoGreen dsDNA reagent (Quant-iT PicoGreen dsDNA; Life Technologies, Gent, Belgium). Total protein was also quantified in the CM fraction utilizing bicinchoninic acid (BCA) as the detection reagent for Cu^+1^, which is formed when Cu^+2^ is reduced by protein in alkaline environment (Micro BCA Protein Assay Kit, Rockford, USA). Results were expressed in μg/mL after 3 days, 21 days and 28 days of culture in text.

### Immunoassays for hyaluronan, aggrecan, IL-6, IL-8, PGE_2_ and MMPs

Hyaluronan (DuoSet ELISA, R&D, Abingdon, UK), IL-6, IL-8, aggrecan, MMP-3 (Enzyme Amplified Sensitivity Immunoassays; Biosource Europe, Merelbeke, Belgium), PGE_2_ (DetectX Prostaglandin E_2_ High Sensitivity immunoassay; Arbor, Michigan, USA) and MMP-1; -2; -9; -13 (Fluorokine MAP, R&D) were measured in different compartments (culture supernatant, FRM and CM) after key intervals throughout the culture time (i.e. 0–3, 17–21, 24–28 days) according to the manufacturer’s recommendations. No cross-reactivity existed between IL-6 and IL-8 assays. Less than 0.5% cross-reactivity and interference was observed between MMPs. The production of hyaluronan and aggrecan were expressed in ng/μg of DNA in text and, respectively, in figure and in table after 3 days, 21 days and 28 days of culture. The production of IL-6, IL-8, and PGE_2_ were expressed in pg/μg of DNA and MMP-3 in ng/μg of DNA after 0–3, 17–21, 24–28 days of culture in table. Results of IL-6, IL-8, PGE_2_ and MMP-3 were also expressed in percentage of production in comparison with alginate considered as control (100%) in table.

### Nitrite assay

The spectrophotometric method based upon the Griess reaction was used to quantify nitrite in different compartments after key intervals throughout the culture time (i.e. 0–3, 17–21, 24–28 days). Griess reagent (0.5% sulphanilamide, 0.05% naphtyl ethylenediamine dihydrochloride, 2.5% H_3_PO_4_) was mixed with 100 μL of conditioned culture medium or sodium nitrite (NaNO_2_, standard solution). The absorption was measured at 540 nm. The NO production was expressed in nmol/μg of DNA in text.

### Statistical analysis

The results are presented as mean (95% confidence interval). Experiments were performed in triplicate and the obtained values were average for the purpose of statistical analysis. Statistical significance was determined using GraphPad Prism software, version 6 for DNA, total protein, hyaluronan, aggrecan, IL-6, IL-8, PGE_2_, and MMPs after one way ANOVA, followed, if positive by the Bonferroni’s multiple comparison post-test and for NO, after Student’s t-test. P-values were considered significant when p<0.05.

## Results

### SEM analysis of the CA beads

Two different techniques, SEM and ESEM, have been performed to observe the ultrastructure of the beads. Classical SEM pictures were the most relevant for illustrating the aspect of the CA beads (Fig 1B–1E). The surface showed microreliefs visible at high magnification (x2000; Fig 1C). The visualization of the internal structure by tearing bead revealed large filaments (about 10 μm thick) with a smooth folded surface, the chitosan trabeculae (Fig 1D), surrounded by a microporous network of thin interconnected filaments (less than 100 nm in thickness) visible at a high magnification (x10000) probably corresponding to the alginate matrix (Fig 1E).

### TEM observation of chondrocytes embedded in CA beads

**Fig 2:**
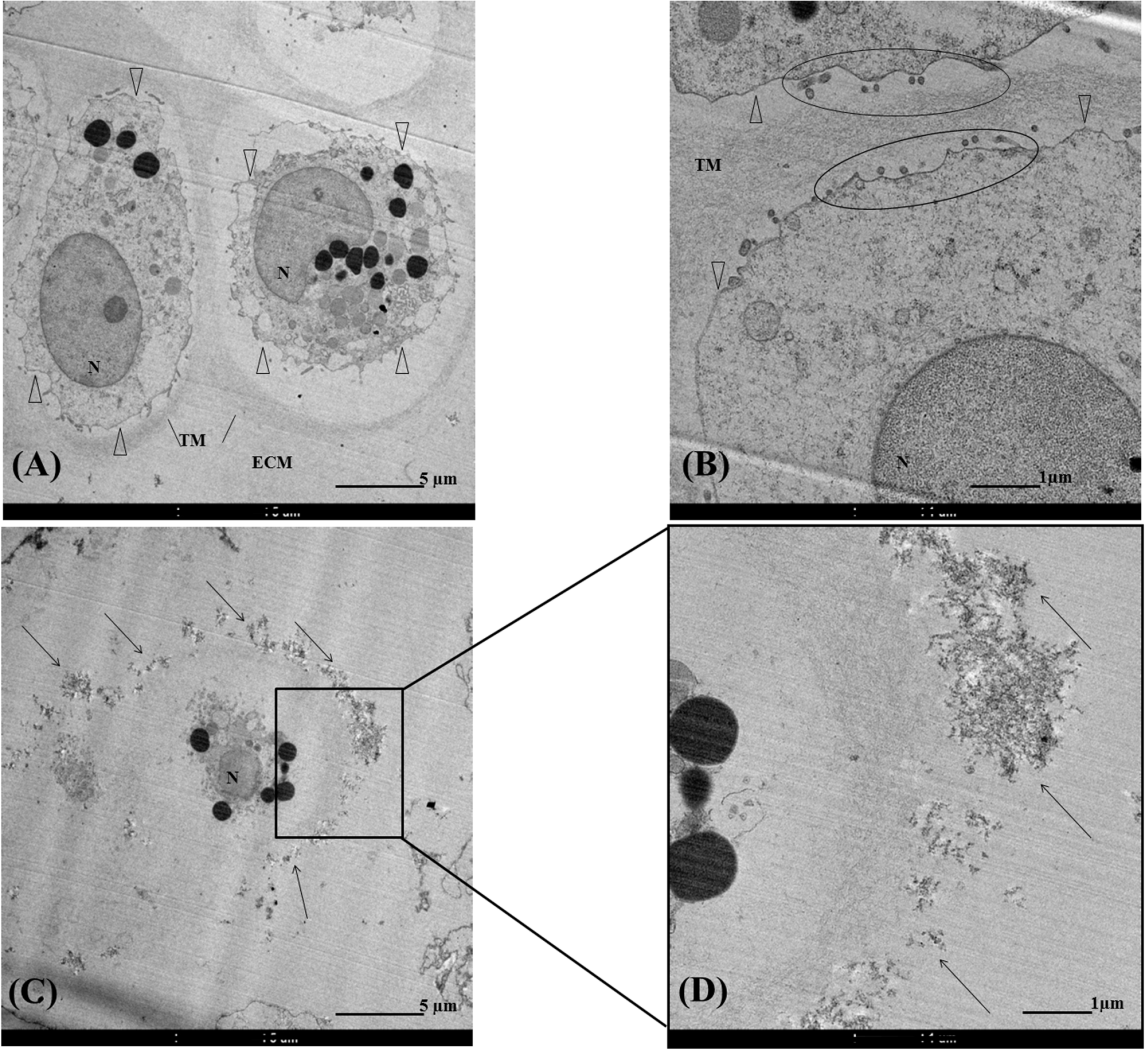
Transmission electron microscopy of chondrocytes in chitosan-alginate beads sections. Transmission electron micrograph of chondrocytes (A-D). N = nucleus. Δ represented pericellular matrix. TM = territorial matrix. ECM = extracellular matrix. Areas surrounded in (B) presented matrix‘s microvillus. Arrow in (C-D) represented flaky accumulation around chondrocyte.

Chondrocytes cultivated in CA beads during 28 days were observed by TEM (Fig 2A–2D). CA beads sections showed mostly single chondrocytes although sometimes small clusters, containing about 3–5 cells, were seen as well dispersed among chitosan trabeculae. A spherical morphology of chondrocyte about 10 μm in diameter with typical matrix organization (Fig 2A–2C) was observed. Pericellular matrix characterized by microvillus with matrix vesicles (Fig 2B) was followed by a fine granules appearance that corresponded to territorial matrix and extracellular matrix. Fig 2C and 2D showed an electron dense flaky accumulation around chondrocytes. An enlargement of an area (Fig 2D) revealed a direct contact between territorial matrix and likely chitosan.

### Histological analysis of the beads

**Fig 3:**
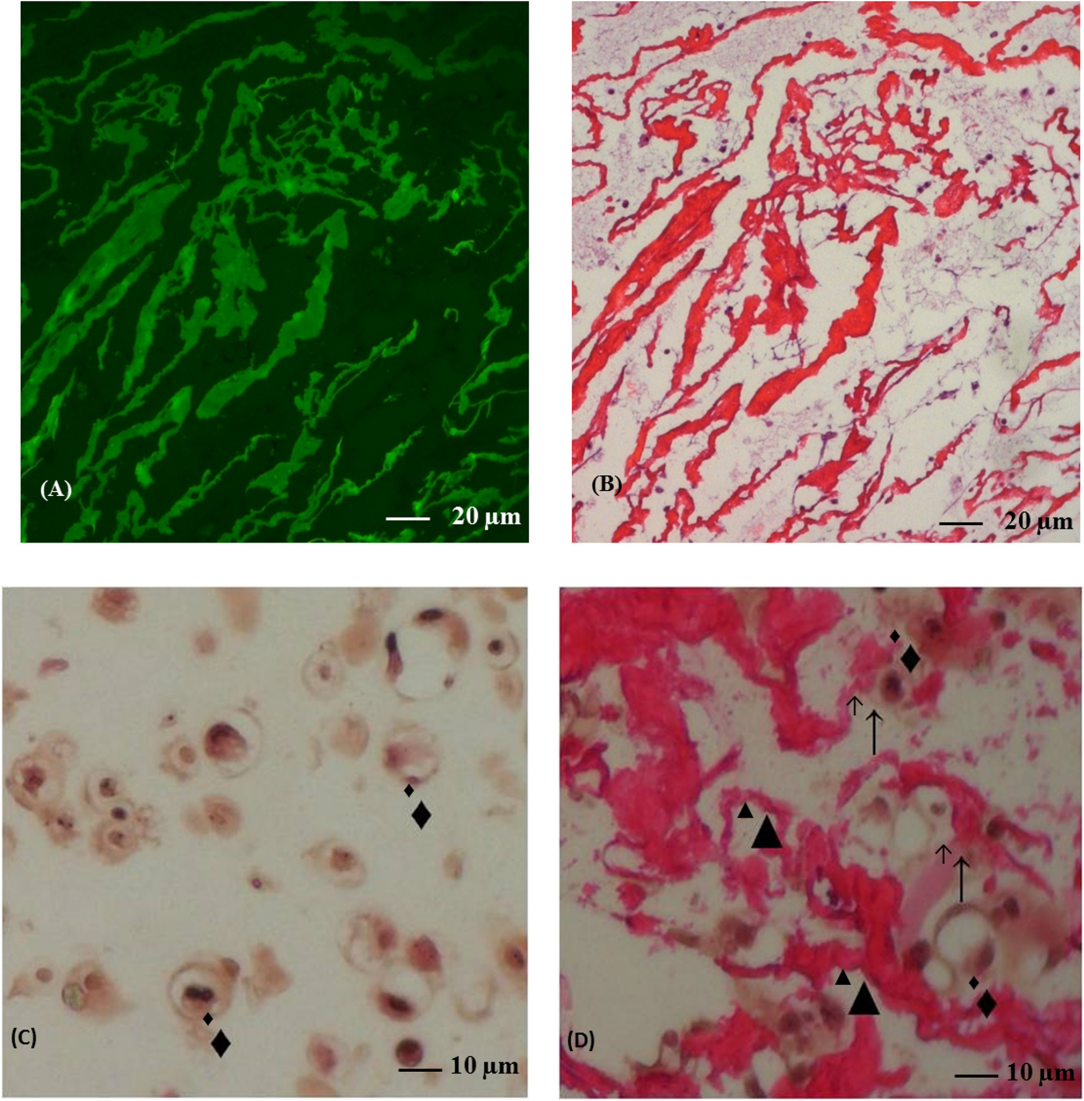
Hematoxylin and eosin stained section from alginate and chitosan-alginate beads. Fluorescence microscopy in chitosan-alginate beads section (A). The same section stained by hematoxylin and eosin (B; original magnification x20). Section in alginate (C) or chitosan-alginate (D) beads stained by hematoxylin and eosin (original magnification x40). Black triangles represented chitosan trabeculae, black diamonds represented chondrocytes and ↑showed contact.

**Fig 4:**
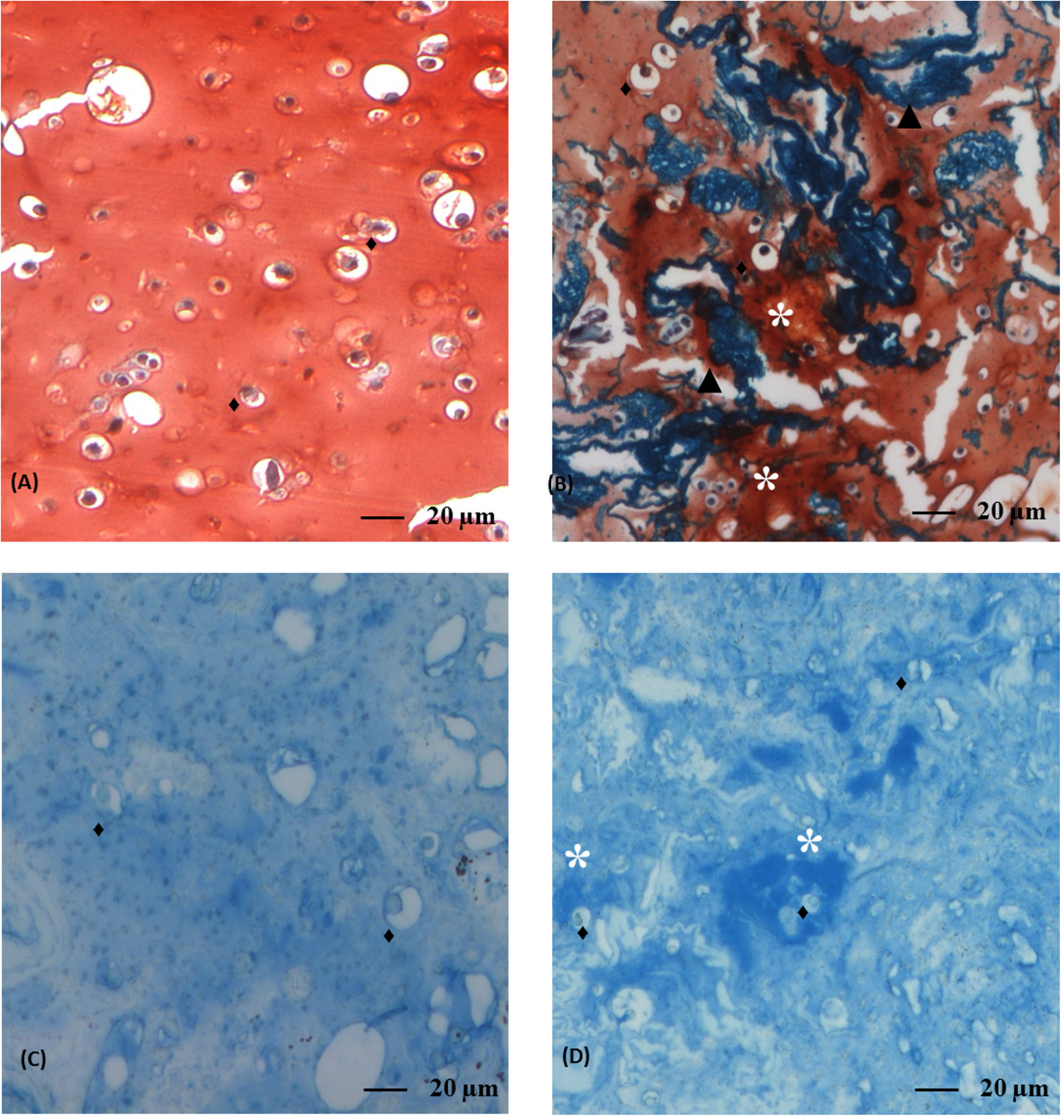
Safranin-O/fast green and alcian blue stained sections from alginate and chitosan-alginate beads. Sections in alginate (A and C) or chitosan-alginate (B and D) beads. Safranin-O/fast green (A and B) or alcian blue staining (C and D). Original magnification x20. Black triangles represented chitosan trabeculae, black diamonds represented chondrocytes and stars indicated GAGs deposition.

To identify chitosan trabeculae, we have labeled chitosan with FITC (Fig 3A). Fig 3B showed that chitosan trabeculae were homogenously distributed in the bead. Hematoxylin-eosin staining revealed that the chondrocytes cultivated for 28 days in A (Fig 3C) or CA (Fig 3D) beads were spherical, located in lacuna, scattered in matrix and isolated from each other as in native articular cartilage. Direct contact between chondrocytes and chitosan trabeculae was observed (Fig 3D). Safranin-O (Fig 4A and 4B) and alcian blue (Fig 4C and 4D) both stained GAGs. After 28 days of culture, a higher amount of GAGs accumulated around chondrocytes in CA beads compared to A beads as revealed by the red staining in Safranin-O (Fig 4B) and the blue staining in alcian blue (Fig 4D).

### DNA and total protein quantification

In CA beads, DNA content in CM fraction remained constant over time (3 days: 5.1 (2.4–7.9) μg/mL vs 21 days: 5.2 (3.2–7.2) μg/mL vs 28 days: 5.4 (3.1–7.6) μg/mL). In contrast, DNA content tended to increase after 21 and 28 days of culture in A beads (p>0.05; 3 days: 4.7 (1.9–7.4) μg/mL vs 21 days: 6.7 (3.3–10.1) μg/mL vs 28 days: 7.1 (4.1–10.1) μg/mL) days in comparison to 3 days. Total protein content in A or CA beads did not change with time (p>0.05; A beads: 3 days: 125.3 (98.3–152.2) μg/mL vs 21 days: 164.3 (136.2–192.3) μg/mL vs 28 days: 166.7 (134.4–199.0) μg/mL; CA beads: 3 days: 127.5 (103.2–151.8) μg/mL vs 21 days: 129.6 (106.7–152.6) μg/mL vs 28 days: 141.6 (106.2–177.0) μg/mL).

### Production of hyaluronan and aggrecan compound

**Fig 5:**
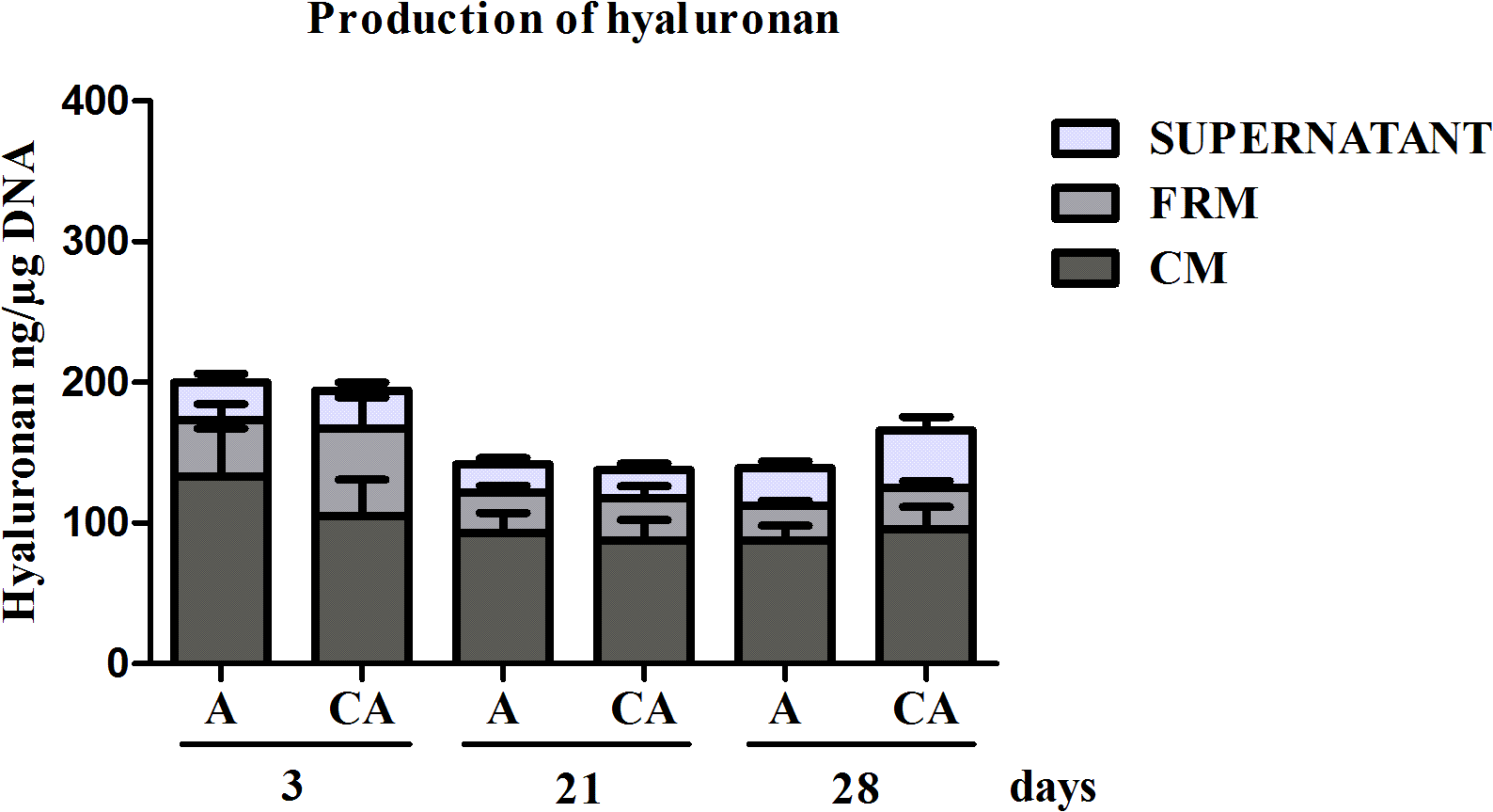
Production of hyaluronan by chondrocytes in alginate and chitosan-alginate beads. Hyaluronan was quantified in CM (cellular matrix), FRM (further-removed matrix) and culture supernatant after key intervals throughout the culture time (i.e. 3, 21, 28 days). Productions were expressed in absolute values (ng) and normalized to DNA content. Results were presented as mean ± SEM of 4 independent experiments (n = 4). Statistical significance was determined after one way ANOVA, followed, if positive, by the Bonferroni’s multiple comparison post-test.

Hyaluronan and aggrecan production was measured in culture supernatant, FRM and CM fractions. Hyaluronan was detected in these three fractions (Fig 5). No significant difference was observed after 3 and 21 days of culture between A (p>0.05; 3 days: 197 (97–297) ng/μg DNA vs 21 days: 142 (94–190) ng/μg DNA) and CA (3 days: 194 (75–313) ng/μg DNA vs 21 days: 137 (92–183) ng/μg DNA) beads. However, hyaluronan tended to increase in CA beads (165 (126–205) ng/μg DNA) in comparison to A beads (139 (118–160) ng/μg DNA; p>0.05) after 28 days of culture (Fig 5). No aggrecan was detected in culture supernatant (data not shown). Nevertheless, aggrecan was detected in both FRM and CM fractions after 3, 21 and 28 days of culture. As reported in Table 1, aggrecan accumulated in the extracellular matrix in both A and CA beads. After 21 days of culture, aggrecan accumulated in the beads was 1.2 fold greater in CA beads compared to A beads (p>0.05) In contrast, no trends were observed after 3 and 28 days (Table 1).

**Table 1:** Production of aggrecan (ng/μg DNA) by chondrocytes in A and CA beads after key intervals throughout the culture time: 3, 21, 28 days.

Beads	Days	FRM	CM	FRM+CM
A	3	615 (604–1,835)	377 (182–936)	992 (778–2,764)
	21	1,490 (900–2,080)	578 (190–966)	2,068 (1,243–2,892)
	28	2,179 (497–4,856)	520 (103–936)	2,699 (333–5,732)
CA	3	481 (332–1,294)	272 (90–635)	753 (469–1,925)
	21	2,036 (381–3,691)	446 (283–609)	2,482 (741–4,223)
	28	2,177 (30–4,384)	387 (271–502)	2,564 (362–4,765)

Values are reported as mean (95% confidence interval) FRM: Further-removed matrix; CM: Cellular matrix; A: alginate; CA: chitosan-alginate

### Production of inflammation mediators

Inflammatory mediators were quantified in all compartments but only detectable in the culture supernatants (data not shown). In comparison with A beads, CA beads significantly reduced the production of IL-6 (p = 0.0012), IL-8 (p<0.0001) and PGE_2_ (p = 0.0056) after 3 days of culture (Table 2). This significant inhibitory effect was sustained during the culture period of 17–21 days for both IL-6 (p = 0.0028) and IL-8 (p<0.0001) and during the culture period 24–28 days for IL-8 (p<0.0001). Moreover, after 3 days of culture, NO production decreased significantly (p = 0.0010) in CA (2.9 (0.2–5.6) nmol/μg DNA) beads compared to A beads (9.5 (4.1–22.4) nmol/μg DNA). No detectable levels of NO were found in culture supernatants after 3 days of culture (data not shown).

**Table 2:** Production of IL-6, IL-8, PGE_2_ and MMP-3 by chondrocytes in A and CA beads after key intervals throughout the culture time: 0–3, 17–21, 24–28 days.

		0–3	17–21	24–28
IL-6	A (pg/μg DNA)	8,153 (4,286–20,592)	695 (95–1,484)	229 (1–460)
	CA (pg/μg DNA)	2,530 (1,689–6,750)	229 (114–573)	147 (99–395)
	% of alginate control	22 (1–43) **	28 (5–63) **	78 (13–169)
IL-8	A (pg/μg DNA)	30,286 (13,757–74,328)	2,404 (116–4,691)	501 (156–845)
	CA (pg/μg DNA)	11,773 (10,964–34,509)	197 (68–463)	108 (18–235)
	% of alginate control	39 (4–74) ***	8 (3–19) ***	21 (2–43) ***
PGE_2_	A (pg/μg DNA)	10,894 (411–22,199)	354 (331–1,040)	105 (7–217)
	CA (pg/μg DNA)	3,153 (261–6,568)	320 (229–869)	84 (32–201)
	% of alginate control	31 (21–41) **	102 (9–195)	82 (36–129)
MMP-3	A (ng/μg DNA)	3,984 (5,002–12,970)	1,727 (1,686–5,140)	460 (109–810)
	CA (ng/μg DNA)	2,328 (3,523–8,179)	498 (59–1,056)	335 (65–605)
	% of alginate control	46 (29–64) **	50 (19–119) **	73 (53–92)

Production of IL-6, IL-8 PGE_2_ and MMP-3 by chondrocytes in A and CA beads. IL-6, IL-8, PGE_2_and MMP-3 were quantified in cell culture supernatants after key intervals throughout the culture time (i.e. 0–3, 17–21, 24–28 days). Productions were normalized to DNA content. Results were expressed as concentration per μg DNA of chondrocyte production in A and CA beads, and as the percentage of chondrocyte production in A beads (100% = control). Results were expressed as mean (95% confidence interval) of 4 independent experiments (n = 4). Statistical significance in comparison to production in A beads (100% = control) was determined after one way ANOVA, followed, if positive, by the Bonferroni’s multiple comparison post-test: A vs CA: *** p<0.001; ** p<0.01.

### MMPs production

MMP-1, -2, -9, -13 productions were not significantly modified by matrix composition and by culture times (data not show). In comparison to A beads, the production of MMP-3 was significantly decreased in CA beads (Table 2) after 3 days of culture (p = 0.0025) and during the 17–21 days of culture period (p = 0.0043). During the period of 24–28 days, the production of MMP-3 in CA beads was lower than the in A beads but the difference was not significant (p>0.05).

## Discussion

This work describes an original method to incorporate cells in chitosan enriched matrix and the influence of this tridimensional matrix on chondrocytes metabolism. This patented method [38] consists of a mixing procedure of alginate alkaline and chitosan acid solution, the gelation of this mixture, and the incorporation of cells in a neutral solution that was suitable to culture. Patent can be found in S1 File.

In this new biomaterial chondrocytes were like in cartilage matrix: scattered, isolated from each other, spherical and surrounded by typical organization zones of the matrix. Moreover, microscopic analysis has revealed that chondrocytes were in contact with chitosan trabeculae especially with territorial matrix. This fact possibly explained the particular behavior of chondrocytes when they are cultured in this biomaterial. Using FITC-labeled chitosan and electronic microscopy, we have observed that chitosan trabeculae formed a network filled by alginate fibers. Taken all together, these results showed that CA beads were an original and interesting biomaterial. Indeed, all methods used so far did not allowed alginate, chitosan and cell to mix. An alginate scaffold surrounded by a shell of chitosan [39, 40] has been developed. In that biomaterial, chitosan and alginate formed two separate phases. Cells were resuspended in alginate solution with no contact between chitosan and cells. Another production method for alginate-chitosan mixed scaffold [41–43] required freezing and freeze drying step that were not relevant and suitable technical procedures for embedded cell culture. Another, particularity of this biomaterial was the non-animal origin of the chitosan. We have used an ultra-pure chitosan extracted from *Agaricus bisporus* mushroom.

This study also demonstrated that the presence of chitosan in the extracellular matrix affected beneficially human chondrocytes metabolism. In summary, chondrocytes cultured in CA beads produced more GAGs but less pro-inflammatory mediators and catabolic factors. Clearly, these findings indicated that chitosan may exert a positive influence on chondrocyte and promote matrix deposition. Indeed, the presence of chitosan promoted the accumulation of GAGs around the cells revealed by blue alcian and safranin O/fast green staining. In addition, using an immunoassay specific for the aggrecan, we have shown that aggrecan contained in the surrounding matrix was higher in CA beads suggesting that CA matrix may accelerate aggrecan deposition in the extracellular matrix. These results confirmed a previous work demonstrating that aggrecan gene expression by chondrocytes cultivated on a pre-coated 4% chitosan coverchip for 7 days was higher than on uncoated coverslips [31]. In contrast, hyaluronan content remained stable whatever the culture condition. Another key result of our study was the fast inhibitory effect of chitosan matrix on the production of inflammatory mediators and catabolic factors. These results are very important in the context of the treatment of OA cartilage defect. Indeed, *in vitro* and *in vivo* studies have shown the major role played by IL-6, IL-8, NO and PGE_2_ in cartilage degradation [44–47] and synovitis onset and progression [48, 49]. In fact, an excessive production of cytokines by the inflamed synovium and activated chondrocytes play an important role in the pathophysiology of OA. IL-1β and TNFα can stimulate their own production and induce chondrocytes and synovial cells to produce other cytokines, such as IL-8, IL-6 and PGE_2_ production [47]. NO is also a factor involved in the IL-1β-mediated catabolic effect and then also participate to cartilage metabolism dysregulation [50]. These data corroborate those of previous studies reporting an anti-inflammatory action of chitosan. Pangestuti et al. demonstrated that chitosan at the concentration of 500 μg/mL in solution inhibited PGE_2_, NO and IL-6 production by murine BV2 microglia cells [51]. Another interesting study [52] showed that chitosan oligosaccharide pretreatment inhibited NO production by HT-29 cells.

An important decrease of MMP-3 production was also observed in the CA beads compared to A beads suggesting an anti-catabolic effect of chitosan. Indeed, MMP-3 was shown to be responsible for the proteolysis of extracellular matrix proteins, mainly aggrecan [53] and of the activation of pro-collagenases [54, 55]. By this way, MMP-3 participates in metalloproteinase cascade activation and in consequence to cartilage degradation [56, 57]. These results are in accordance with those of Liu et al. [58] showing that 2% of carboxymethylated chitosan solution injected in rabbit knee significantly decreased MMP-3 mRNA cartilage expression 6 weeks after injection. More surprising was the absence of CA effects on the other MMPs. This could be explained by a specific binding between chitosan matrix and MMP-3 limiting MMP-3 release in the culture supernatant or by a specific action of CA matrix on MMP-3 signaling pathway. However, these hypotheses remain highly speculative and should be investigated in deep.

One limitation of this study was the absence of chitosan beads alone as control. The reason is that it was not possible to prepare chitosan beads with embedded chondrocytes that were suitable to culture. Indeed, chitosan solution must be prepared by chitosan powder dissolution in acetic acid. Chondrocytes were then suspended in this acid solution that was no appropriate with cells viability.

These results suggest that the CA beads could be used as cell carrier to repair traumatic or degenerative cartilage lesion. Indeed, if implanted in cartilage lesion, chitosan-enriched biomaterial could promote cartilage matrix synthesis by chondrocytes but also decrease the local production of inflammatory and catabolic factors. These multiple biological activities confer to chitosan a benefit compared to other biomaterials. For example, a matrix composed with collagen or with hyaluronic acid failed to decrease pro-inflammatory and pro-catabolic mediators synthesis by chondrocytes [59, 60].

## Conclusions

CA beads could be used as carrier for cell transplantation, particularly to repair degenerative cartilage defect. This *in vitro* test highlighted beneficial and promising effects of this new biomaterial on human OA chondrocytes. They produced less inflammatory and catabolic mediators and maintained the synthesis of cartilage specific matrix components. All these biological activities are beneficial for cartilage repair.

## Supporting Information

S1 File Patent entitled "Cell cultivation in chitosan alginate hydrogel beads". (PDF)
