# Supplementary material for: Chitosan Enriched Three-Dimensional Matrix Reduces Inflammatory and Catabolic Mediators Production by Human Chondrocytes
Source: PLoS One. 2015 May 28;10(5):e0128362. doi: 10.1371/journal.pone.0128362 (PMC4447380; doi:10.1371/journal.pone.0128362)
Supplement: S1 File — (PDF) [file pone.0128362.s001.pdf]

(12) INTERNATIONAL APPLICATION PUBLISHED UNDER THE PATENT COOPERATION TREATY (PCT)

(19) World Intellectual Property Organization
International Bureau

(43) International Publication Date
1 September 2011 (01.09.2011)

PCT

(10) International Publication Number
WO 2011/104131 A1

(51) International Patent Classification:
A61L 27/20 (2006.01) *A61L 27/38* (2006.01)

(21) International Application Number:
PCT/EP2011/052013

(22) International Filing Date:
11 February 2011 (11.02.2011)

(25) Filing Language: English

(26) Publication Language: English

(30) Priority Data:
10154712.3 25 February 2010 (25.02.2010) EP

(71) Applicant *(for all designated States except US)*: **UNIVERSITE DE LIEGE** [BE/BE]; Interface Entreprise-Université, Antheunis, Nicole, avenue Pré-Aily, 4, B-4031 Angleur (BE).

(72) Inventors; and
(75) Inventors/Applicants *(for US only)*: **HENROTIN, Yves** [BE/BE]; Aux Grands Champs 63, B-4052 Beaufays (BE). **KESTELOOT, Frédéric** [BE/BE]; Rue Hasquette 17, B-4540 Ampsin (BE). **SANCHEZ, Christelle** [BE/BE]; Sur les Charneux 382, B-4870 Fraipont (BE).

(74) Common Representative: **UNIVERSITE DE LIEGE**; Interface Entreprise-Université, Antheunis, Nicole, avenue Pré-Aily, 4, B-4031 Angleur (BE).

(81) Designated States *(unless otherwise indicated, for every kind of national protection available)*: AE, AG, AL, AM, AO, AT, AU, AZ, BA, BB, BG, BH, BR, BW, BY, BZ, CA, CH, CL, CN, CO, CR, CU, CZ, DE, DK, DM, DO, DZ, EC, EE, EG, ES, FI, GB, GD, GE, GH, GM, GT, HN, HR, HU, ID, IL, IN, IS, JP, KE, KG, KM, KN, KP, KR, KZ, LA, LC, LK, LR, LS, LT, LU, LY, MA, MD, ME, MG, MK, MN, MW, MX, MY, MZ, NA, NG, NI, NO, NZ, OM, PE, PG, PH, PL, PT, RO, RS, RU, SC, SD, SE, SG, SK, SL, SM, ST, SV, SY, TH, TJ, TM, TN, TR, TT, TZ, UA, UG, US, UZ, VC, VN, ZA, ZM, ZW.

(84) Designated States *(unless otherwise indicated, for every kind of regional protection available)*: ARIPO (BW, GH, GM, KE, LR, LS, MW, MZ, NA, SD, SL, SZ, TZ, UG, ZM, ZW), Eurasian (AM, AZ, BY, KG, KZ, MD, RU, TJ, TM), European (AL, AT, BE, BG, CH, CY, CZ, DE, DK, EE, ES, FI, FR, GB, GR, HR, HU, IE, IS, IT, LT, LU, LV, MC, MK, MT, NL, NO, PL, PT, RO, RS, SE, SI, SK, SM, TR), OAPI (BF, BJ, CF, CG, CI, CM, GA, GN, GQ, GW, ML, MR, NE, SN, TD, TG).

Declarations under Rule 4.17:
— *as to applicant's entitlement to apply for and be granted a patent (Rule 4.17(ii))*

Published:
— *with international search report (Art. 21(3))*

(54) **Title**: CELL CULTIVATION IN CHITOSAN ALGINATE HYDROGEL BEADS.

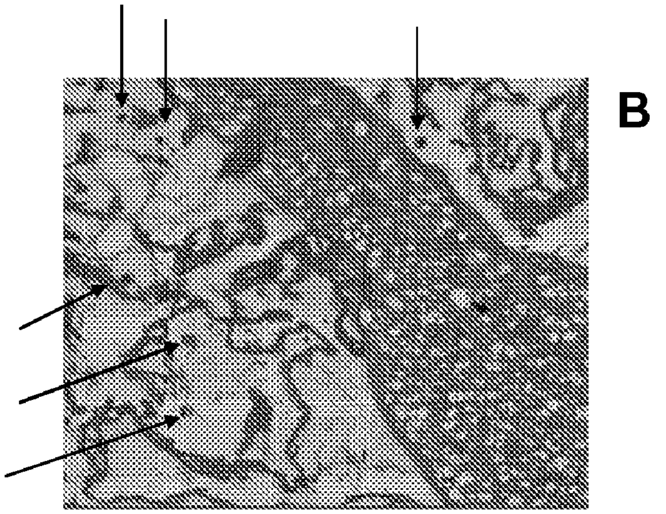

Figure 2

(57) **Abstract**: The present invention relates to a method of producing a hydrogel matrix comprising cartilage-forming cells wherein alginate, chitosan and cartilage- forming cells are mixed and subsequently polymerised into beads.

WO 2011/104131 A1

CELL CULTIVATION IN CHITOSAN ALGINATE HYDROGEL BEADS

Field of the invention

The present invention relates to scaffolds for the cultivation of cartilage-forming cells, more particular chondrocytes. The present invention further relates to cellularised beads for implantation. The present invention further relates to methods and tools for cartilage repair.

Background of the invention

Isolated and expanded cartilage-forming cells have been used for implantation since several decades. The in vitro expansion to obtain a sufficient amount of cells however may lead to cells which upon implantation result in cells death, scar tissue or hyperdifferentiated cells. This is especially the case for cartilage repair where chondrocytes culture, upon implantation often produces fibrous or bone-like material instead of the envisaged hyaline cartilage.

Attempts have been made to cultivate chondrocyte cells in a three dimensional matrix wherein the extracellular matrix of cartilage is mimicked. A variety of gels has been used wherein cell are cultivated. For example, the prior art discloses beads with a core of alginate and a coating of chitosan (Babister et al. (2008) *Biomaterials* **29**, 58-65).

Methods to prepare hydrogel beads can be divided in three groups. A first type relates to porous matrices which are populated by cells. Herein the matrices have a large pore size to allow the migration of cells into the matrix. Li *et al.* (2008) *J. Biomed. Mater. Res. A.* **86**, 552-559, describe a porous scaffold containing 2.4 % alginate/2.4 % chitosan which is subsequently seeded with cells.

As the cells migrate from the outside into the interior of the matrix, these matrices often have many cells at the outer cells and a low amount of cells at the interior.

In a second type of methods cells are mixed with a constituent of the matrix whereafter the matrix is being formed. Bernstein *et al.* (2009) *Biotechnol. Prog.*

25, 1146-1152, describe methods wherein beads with 2.5 % alginate and 1.4 % chitosan are formed. The cartilage therein is described as being of low quality.

In a third method, as illustrated in WO2007/135114, cells are encapsulated in a gelling solution obtained from an aqueous solution of a mixture of a polysaccharide such as alginate and an oligosaccharide derivative of chitosan highly branched. The aqueous solution is gelled with gelling agents with the aim to encapsulate the cells.

In each type of methods the choice of hydrogel has a strong impact on the phenotype of the cells, and subsequently on the quality of the implant. Particularly in the hydrogel of WO2007/135114 , oligosaccharide derivative of chitosan has a degree of derivatization of at least 40% and the polysaccharide mixture or alginate mixture has an ionic strength which is not optimal for keeping cells alive. Further this 3D matrix fails to keep chondrocyte phenotype stable as shown by an increase of cell proliferation and a decrease of aggrecan synthesis.

There remains a need for further improvements in the selection of matrix material and in the methods to produce cellularised implants.

Summary of the invention

One aspect of the present invention relates to methods of producing a hydrogel matrix comprising cartilage-forming cells comprising the steps of:

- providing a solution of alginate,
- providing a solution with chitosan having Mw lower than 60 kD,
- mixing the solution of alginate and the solution of chitosan with cartilage-forming cells, wherein the mixed solution comprises between 0.5 and 0.7 % (w/v) of chitosan and between 1 and 1.4 % alginate (w/v),
- introducing droplets of the mixed solution into a solution with Ca^{2+} or Sr^{2+} cations and
- isolating gellified beads from the solution with cations.

In certain embodiments of methods of the present invention the droplets of the mixed solution are introduced in a solution Sr^{2+} ions.

In particular embodiments of this method the mixed solution comprises 0.6 % chitosan and or comprises 1.2 % alginate.

In particular embodiments of methods of the present invention the ratio between alginate and chitosan in the mixed solution is between 1.4 and 2.8, or between 1.75 and 2.25 or is about 2.

In particular embodiments of methods of the present invention the chitosan has a Mw of between 35 and 45 kD and/or is of animal or preferably of vegetable origin.

In other particular embodiments of methods of the present invention the method further comprises the step of cultivating the beads comprising cartilage-forming cells in a growth medium. Such a cultivation can be performed for up to 7, 14, 21 or even 28 days.

In other particular embodiments of methods of the present invention the growth medium comprises serum.

In other particular embodiments of methods of the present invention the formation of beads is performed by passing droplets through a needle to obtain a particle with a diameter between about 0.2 and 5 mm.

In other particular embodiments of methods of the present invention, the method further comprises the step of mixing the cultivated beads in a thermosensitive hydrogel. Herein the ratio between beads and hydrogel is for example between 5/1 and 1/1, or between 4/1 and 2/1.

Another aspect of the present invention refers to a spherical hydrogel bead comprising a homogeneous mixture of chitosan and alginate and further comprising cartilage-forming cells, wherein said bead is obtainable by the methods as described above.

Another aspect of the present invention refers to a spherical hydrogel bead with a diameter between 0.01 and 5 mm, comprising a homogeneous mixture of chitosan and alginate and further comprising cartilage-forming cells within the matrix, characterised in that the bead comprises between 1 and 1.4 % alginate and between 0.5 and 0.7 % chitosan, for example, the bead comprises 1.2 % alginate, or for example the bead comprises 0.6% chitosan.

In particular embodiments of beads of the present invention the ratio of alginate/chitosan is between 1.4 and 2.8, or between 1.75 and 2.25, or between 1.8 and 2.2.

In other particular embodiments of beads of the present invention the chitosan has a Mw between 35 and 45 kD.

In other particular embodiments of beads of the present invention the cartilage-forming cells have a concentration of between 50000 or 60000 to 100000 or 150000 cells per bead.

In other particular embodiments of beads of the present invention the cartilage-forming cells, after 21 days of cultivation, express below 0.5 pg lactate dehydrogenase (LDH), or the cartilage-forming cells, after 21 days of cultivation express below 0.02 units alkaline phosphatase per ng LDH, when grown in the presence of serum.

Another aspect of the present invention relates to the use of cartilage-forming cells comprised in a bead as described or produced as described above for the repair of cartilage defects, such as osteoarthritis.

Another aspect of the present invention relates to the use of cartilage-forming cells comprised in a bead as described or produced as described above for the manufacture of a medicament for the repair of cartilage defects.

Advantages of beads as prepared in the present invention and their use:

In prior art cartilage transplantation methods, a low number of autologous chondrocytes is harvested from cartilage biopsies coming from normal area of cartilage, and then cultured in monolayer for proliferation until to get an optimal number of cells for grafting. When they are cultured in monolayer, chondrocytes progressively loose their phenotype, and fail to a cartilaginous extracellular matrix, and become fibroblasts which mainly produce scar tissue. To avoid this dedifferentiating process or to promote cell redifferentiation after monolayer culture, chondrocytes can be cultured in a three dimensional matrix, such as alginate beads. However, in alginate beads, chondrocytes undergo a hypertrophic differentiation and mineralize the surrounding matrix. Hypertrophic differentiation and matrix mineralization are undesirable effects associated with

osteoarthritis. The present invention demonstrates that, in contrast to chondrocytes cultured in alginate beads, chondrocytes cultured in chitosan/alginate beads do not differentiate in hypertrophic chondrocytes. The beads which are in use in accordance with the present invention stabilize the chondrocyte phenotype. Indeed, after several weeks of culture in the presence of serum e.g. 10 % FBS (which is a condition supporting the hypertrophic differentiation of the chondrocytes), chondrocytes cultured in chitosan/alginate beads between 21 to 28 days produce marginal amounts of alkaline phosphatase (AP) which is a specific marker of hypertrophy, compared to chondrocytes cultured in alginate beads (figure 4).

Chondrocytes cultured in chitosan/alginate beads produced significantly higher amounts of the cartilage-specific molecule aggrecan, than chondrocytes in alginate beads and significantly less pro-inflammatory (IL-6, nitric oxide) and catabolic factors (stromelysine-1). The chitosan/alginate mixture described in the present invention prevents the hypertrophic differentiation of chondrocytes as illustrated by the decrease in alkaline phosphatase expression.

This particular effect indicates that chitosan/alginate beads are potential carriers for cells transplantation, and particularly to repair tissue, including cartilage defect.

Brief description of the figures

Figure 1 shows a section of an alginate bead (A) and a chitosan/alginate bead (B) [chitosan: dark grey trabeculae; alginate: light grey background].

Figure 2 shows at low magnification (A) chitosan/alginate beads embedded in a chitosan hydrogel. At higher magnification (B) chondrocytes are visible (indicated by arrows).

Figure 3 shows the expression of Interleukin-6 (Il-6) (represented as nanogram IL-6 / per ng lactate dehydrogenase) at different time points. Figure 3A Left bar: cells cultivated in calcium alginate; middle bar: cells cultivated in chitosan (20 kD) / calcium alginate beads; right bar: cells cultivated in chitosan (40 kD) / calcium alginate beads. (chitosan/alginate ration is 1/2). Figure 3B shows IL-6

production in chondrocytes after 9 days of cultivation in alginate (left) or chitosan/alginate (right) beads using Ca^{2+}, Sr^{2+} or a mixture of Ca^{2+} and Sr^{2+}.

Figure 4 shows the expression of alkaline phosphatase (AP) in alginate beads (closed bars) and in 0.6 % chitosan/ 1.2% alginate beads (open bar) [chitosan 40 kD] in different media [ITS: medium comprising Insulin, transferrin and selenious acid; UG: Ultroser G; FBS fetal bovine serum). (AP concentrations are presented as Units alkaline phosphatase / per ng lactate dehydrogenase) Panel A shows values after 21 days of cultivation, Panel B shows values after 28 days of cultivation.

Figure 5 shows the expression of aggrecan (represented as nanogram aggrecan/per ng lactate dehydrogenase). Figure 5A shows the expression after 13 days of cultivation in alginate beads (left) chitosan (20 kD) / alginate beads (middle) and chitosan (40 kD) / alginate beads (chitosan/alginate ration is 1/2). Figure 5B shows aggrecan production in chondrocytes after 9 days of cultivation in alginate (left) or chitosan/alginate (right) beads using Ca^{2+}, Sr^{2+} or a mixture of Ca^{2+} and Sr^{2+}. CM: Cell-associated Matrix; FRM: Further-Removed matrix*;

Figure 6 shows the expression of MMP-3 (represented as nanogram MMP-3 / per ng lactate dehydrogenase) at different time points. Left bar: cells cultivated in calcium alginate; middle bar: cells cultivated in chitosan (20 kD) / calcium alginate beads; right bar: cells cultivated in chitosan (40 kD) / calcium alginate beads. (chitosan/alginate ratio is 1/2).

Figure 7 shows the implantation of a thermosensitive chitosan hydrogel comprising 0.6 % chitosan/1.2 %alginate beads (*A. bisporus* chitosan (40 kD))

Figure 8 shows the histological evaluation of the implant 15 days after implantation.

A: Subchondral Bone; B: Cell-colonized hydrogel; C: cell colonized Chitosan/alginate bead; D: Cell embedded in chitosan lacunae.

Detailed description of the invention

One aspect of the present invention relates to a method of producing a hydrogel matrix comprising cartilage-forming cells. In this method cells are included in the

gel constituents upon formation of the hydrogel matrix. This method comprises the following steps:

- providing a solution of alginate,
- providing a solution with chitosan of Mw lower than 60 kD,
- mixing the solution of alginate and the solution of chitosan with cartilage-forming cells, wherein the mixed solution comprises between 0.5 and 0.7 % (w/v) of chitosan and between 1 and 1.4 % alginate,
- introducing droplets of the mixed solution into a solution with Ca^{2+} or Sr^{2+} ions
- isolating gellified beads from the solution with cations.

The hydrogel which is obtained by this method results in a homogeneous matrix of calcium alginate and chitosan, wherein the cell are equally distributed throughout the matrix.

The matrix as obtained in the present invention differs from prior art matrices which have a core of one component, coated with a layer of another component.

The matrix as obtained in the present invention has the advantage that the porosity of the matrix can be more accurately defined compared to matrices which are first lyophilised to obtain a certain degree of porosity.

The matrix as obtained in the present invention has the advantage to be composed with a low molecular weight chitosan (lower than 60 kDA) which spontaneously forms an homogenous network in the alginate matrix.

The matrix as obtained in the present invention, wherein cells are included into the matrix upon gelification, has the advantage that the cells are homogeneously distributed over the matrix beads. Porous beads which are seeded afterwards with cells typically will create a gradient of cells whereby the interior core of the matrix contains less cells than the exterior shell of the beads. This often results in cell populations wherein the cells at the core have different properties than those at the outside.

As indicated in the examples section, the alginate and chitosan which is used to prepare the hydrogels is dissolved is strong alkaline or acidic buffers which have a sterilising effect . This is an additional advantage of the Invention .

In the methods in accordance with the present invention the alginate and chitosan solutions can be mixed to obtain beads with different concentrations. Particular embodiments of the present invention relate to beads wherein the composition, prior to the gelification by calcium or strontium ions comprises 0.4, 0.45, 0.5, 0.55, 0.60, 0.65, 0.70, 0.75 or 0.80 % (w/v) chitosan, and independently thereof comprises 0.9, 0.95, 0.1, 0.105, 0.11, 0.115, 0.12, 0.125, 0.13, 0.135, 0.14, 0.145 or 1.5 % (w/v) alginate. In particular embodiments the concentration of chitosan ranges from 0.5 to 0.7 %, or from 0.55 to 0.65 %. In other particular embodiments the concentration of alginate ranges from 1. to 1,4 % or from 1,25 to 1,35 %. A particular embodiment of hydrogel comprises about 0.6 % chitosan and about 1.2 % alginate.

Further embodiments of methods and compositions of the present invention relate to hydrogel composition and beads obtained thereof wherein the ratio between alginate and chitosan in the mixed solution is between 1.4 and 2.8, more particularly between 1.5 and 2.7, more particularly between 1.6 and 2.6, or between 1.75 and 2.25. Particular values of this ratio are about 1.9, 1.95, 2.0, 2.05 and 1.

In the methods of the present invention the average size of the beads can be adapted and empirically determined by adjusting the diameter of the needle which is used to form the droplets which are introduced into the calcium or strontium solution. Envisaged herein are beads with a diameter between 0.01 and 5 mm. These dimensions provide a compromise between the ease of manipulation and the diffusion of nutrients into the beads.

The experiments of the present invention indicate that the physical properties of the chitosan may contribute to the phenotype of the cartilage-forming chondrocytes. Chitosan has been isolated from different animal sources such as crustaceans (prawn shells) or squids. Alternatively the chitosan is of vegetal, more of particularly fungal origin, such as *Mucoralean* strains, *Mucor*

racemosus and *Cunninghamella elegans, Gongronella butleri, Aspergillus niger, Rhizopus oryzae, Lentinus edodes, Pleurotus sajo-caju, Zygosaccharomyces rouxii, Candida albicans* or *Agaricus bisporus.*

Chitosan further exists in diverse types of molecular weight. Herein, the chain length of chitosan may contribute to the three dimensional structure of the hydrogels. Typical chitosans, for use in the present invention has an average molecular weight lower than 60kD particularly between 15 and 50 kD, more particularly between 35 and 45 kD.

Beads which are produced in accordance with the present invention comprise cartilage-forming cells and are cultured to obtain the desired amount of cells for further purposes, such as implantation. The cultivation period can depend from certain factors such as cell type and initial concentration in the bead.

The beads of the present invention are prepared together with chondrocytes, chondrocyte precursors, cartilage-forming mesenchymal stem cells or cartilage-forming stem cells or with mixtures of chondrocytes and cartilage-forming precursor or stem cells.

It has been found that freshly isolated chondrocytes can be cultivated in the beads of the present invention for 7, 14, 21, or 28 days or even longer while maintaining their property to generate hyaline cartilage upon implantation.

The appropriate time of cultivation for the other above mentioned cell types is determined experimentally upon analysis of markers for chondrocyte stability which are described in the examples section of the present invention.

The chitosan/alginate beads of the present condition allow the cultivation of cells in conditions which are described as detrimental for the cultivation of chondrocytes, more particularly allow the growth of chondrocytes in a medium comprising 5, 10, 15 % (volume serum/volume growth medium) serum of foetal or adult animals and overcomes the use of specialised synthetic media for chondrocyte cultivation.

The method of manufacturing beads as described above results in the formation of spherical hydrogel beads which comprise a homogeneous mixture of chitosan and alginate and further comprise cartilage-forming cells which are also homogenously distributed in the matrix of a bead.

Compared to other types of cellularised beads, the bead of the present invention can be prepared in a more reproducible ways than beads which are freeze-dried to provide cavities for the entry and cultivation of cells.

The superior properties of the cells which are cultivated in beads which are prepared in accordance with the methods of the present invention are demonstrated by the limited expression of cell death markers such as lactate dehydrogenase (LDH), the limited expression of inflammation markers, such as interleukins, the limited expression of markers which are typical for bone forming cells, such as alkaline phosphatase and the enhanced expression of cartilage markers such as aggrecan (Figure 5). The expression of markers which are relevant for assessing the phenotypic stability of the chondrocytes have been experimentally determined by cultivating cells in chitosan/alginate beads of the present invention and in alginate beads, in the presence of serum.

It has been further observed that the type of cation that has been used in gelification has an effect on the expression of at least aggrecan and interleukin-6. Herein, the use of strontium instead of calcium decreases as well the expression of IL-6 a marker for inflammation as of aggrecan, a marker for ECM production.

In view of the surprising properties of cartilage-forming cells, grown in beads as prepared in the present invention, one aspect of the invention relates to the use of cartilage-forming cells comprised in a bead as prepared in the present invention for the treatment of cartilage defects, more particularly osteoarthritis.

Depending on the type of cartilage defect, the beads can be implanted shortly after formation of the beads, such that the cells will mainly grow after implantation. Alternatively the beads are implanted after in vitro cultivation for several days or weeks.

In particular embodiments, beads with chondrocytes are formulated as a biphasic implant material formed by beads and gel. This implant includes a polymeric matrix (“hydrogel”), and the spherical three-dimensional beads comprising chitosan and alginate and cells.

This allows to formulate an injectable gel which upon implantation, ensures an optimal space distribution of cells (chondrocytes or others) in the host tissue or

organ. The injectable gel which is typically used herein is a thermo-sensitive gel. These gels remain liquid at ambient temperature (in a device used for introduction in to the patient), but become a solid gel upon introduction into the body at about 37°C. This in situ gelification maintains the beads (and the cells therein) in their spatial distribution. In vitro tests have demonstrated that beads containing cells were homogenously distributed in such a hydrogel when heated to 37°C. Examples of thermosensitive hydrogels include poly(N-isopropylacrylamide (PNIPAAm). A particular type hereof is chitosan.

Without being bound by theory it is believed that the chitosan network within the microbeads confers particular mechanical properties at the beads such that they are less compressible and more resistant to pressure than the beads made of alginate only. The matrices of the present invention provide inter-connected chitosan trabeculae within an alginate gel, resulting in an environment which is favourable for cellular culture by providing an aqueous medium with a neutral pH.

Such trabeculae are obtained by insoluble chitosan which forms coacervates which create a basket-like structure network or trabeculae when mixed with alginate. The trabeculae are of varying thickness and length which provides to the beads particular biological and mechanical properties such as phenotye stabilisation, deformability, elasticity and compressive modulus.

Examples

Example 1. Isolation of chondrocytes

Fragments of cartilage are collected by biopsy and then submitted to three successive enzymatic treatments (hyaluronidase 0.5 mg/ml during 30min at 37°C; pronase 1 mg/ml during 1h at 37°C; collagenase 0.5 mg/ml during 1 night at 37°C in the presence of 1 % Ultroser G) to remove the extracellular matrix. (Ultroser G is a serum substitute of Pall Corporation). After passage on a cells strainer, the chondrocytes are rinsed and centrifuged. The cell pellet is collected and suspended in the chitosan/alginate solution.

Example 2. Preparation of alginate/chitosan beads

Beads are prepared from a homogeneous mixture of chitosan (0.6 % final) and alginate (1.2 % final). The two solutions are prepared separately before being mixed. The solutions of alginate and chitosan are prepared in the following way: An alginate solution 2.4 % (W/v) in 0.16 M NaOH and a solution of chitosan 1.333 % (w/v) in 1.666 M HAc are prepared. To 10 volumes of the alginate solution, 1 volume of a 1 M Hepes solution is added. After homogenization, we 9 volumes of chitosan solution are progressively added, while mixing regularly and vigorously.

Hereafter a cellular pellet of chondrocytes was administered and cautiously dispersed in the chitosan/alginate solution at a concentration of 6 x 10^6 cells/ml. The chitosan/alginate solution with the cells was slowly passed through a 25 gauges needle in a dropwise fashion into a 102 mM $CaCl_2$ solution (Sigma-Aldrich, Bornem, Belgium). After instantaneous gelation, the beads were allowed to gellify further for 10 min in this $CaCl_2$ solution. In the gelled beads, the chondrocytes are distributed in a homogeneous way. At microscopic scale, the chitosan (stained in red by the eosin) forms a basket-like structure, composed of trabeculae or fibres of varying thickness and length (see figure 1 and 2). The interstices herein are filled by alginate (hematoxylin stained in violet) which contains the chondrocytes.

Example 3. Cultivation of chondrocytes in alginate/chitosan beads.

The gelled beads comprising chondrocytes, were washed with a saline solution. Beads were cultured in a 24-well plates by providing 10 beads in one ml of culture medium [DMEM supplemented with 1 % ITS+ (ICN Biomedicals, Asse-Relegem, Belgium), 10 mM HEPES, penicillin (100 U/ml) and streptomycin (100 U/ml), 200 µg/ml glutamine (Biowhittaker Europe, Verviers, Belgium), 50 µg/ml ascorbic acid (Sigma-Aldrich, Bornem, Belgium), 2 mM proline (Gibco, Merelbeke, Belgium)]. (ITS+ is a premixed cell growth system containing in one ml: 0.625 mg insulin, 0.625 mg transferrin, 0.625 µg selenious acid, 0.125 g bovine serum albumin and 0.535 mg linoleic acid).

Example 4. Characterisation of chondrocytes

Chondrocytes in figure 2B were stained with hematoxylin/eosin.

Aggrecan and IL-6 were quantified with ELISA. (Biosource Europe)Alkaline Phosphatase was quantified with a spectrophotometric assay. Briefly, Fifty µl of cell extract were incubated with 100 µl of p-nitrophenylphosphate (K*EM*-EN-TEC, Kobenhavn, Denmark). In the presence of AP, p-NPP is transformed to p-nitrophenol and inorganic phosphate. p-nitrophenol absorbance is measured at 405 nm. A standard preparation of p-nitrophenol was used for calibration. Results were expressed in nmoles of p-nitrophenol released per min and per µg of DNA. One unit AP is defined as one nmole of p-nitrophenol liberated per min.

Lactate dehydrogenase was quantified by testing its enzymatic activity in the culture supernatant. 100 µl of the supernatant or dilutions of standard solution (LDH from rabbit muscle) were mixed with 50 µl of Tris buffer (10 mM Tris-HCl (pH 8.5), 0.1% BSA) containing 800 mM lactate. 100 µl of colorimetric reagent (1,6 mg/ml iodonitrotetrazolium chloride (Sigma-Aldrich, Bornem, Belgium), 4 mg/ml nicotinamide adenine dinucleotide (Roche Diagnostics, Brussels, Belgium), 0.4 mg/ml phenazine methosulfate (Sigma-Aldrich, Bornem, Belgium) was added and the absorbance at 492 nm was read after 10 min of incubation at room temperature.

These data show that chondrocytes which are cultivated in the matrix of the present invention show a number of superior properties:

- cells cultivated in chitosan/alginate show less apoptosis or necrosis compared to alginate beads as indicated by the measurement of lactate dehydrogenase.
- cells cultivated in chitosan/alginate show less signs of inflammation. Chondrocytes cultivated in chitosan/alginate beads do not produce or produce very little amounts of IL-6, IL-8 and NO. Figure 3 shows a dramatic reduction of IL-6 production during the culture of the chondrocytes in mixed beads (alginate/chitosan) compared to alginate beads.

IL-1β is a very active cytokine in the inflammatory reaction and the associated tissue proteolysis. The stimulating effects of IL-1β on the production of

mediators of inflammation or of proteolytic enzymes are less important in alginate/chitosan beads than in alginate beads.

- Chondrocytes cultivated in chitosan/alginate show less signs of catabolic events as indicated by lower production of a matrix metalloproteinase MMP-3, which is involved in the cartilaginous matrix degradation compared to chondrocytes cultivated in alginate beads.

-The growth of chondrocytes in the chitosan/alginate beads has a beneficial and stabilising effect on the chondrocyte phenotype.

Example 5. Formulation of beads in a thermosensitive hydrogel.

Culture medium is removed by per aspiration, and the beads are mixed with a vegetable (*Agaricus bisporus*) chitosan hydrogel (Kitozyme, Alleur, Belgium]. This step is performed below 27°C to avoid hydrogel gelification. A ratio of beads/hydrogel of 3/1 (v/v) has been used.

Example 6. Effect of cations on chondrocyte phenotype.

Figure 3B and 5B show the effect of the choice of cation used in the hydrogel formation. Whereas the choice of calcium or strontium has no effect on the expression of the markers interleukin-6 and aggrecan in beads which consist entirely of alginate, a significant effect is observed when chitosan/alginate are formed with either calcium or strontium ions.

Example 7. Implantation in an animal model

A gel as described under example 5, without chondrocytes, has been implanted in a cartilage defect in a rabbit joint (Figure 7). After 15 days of implantation, the implant was evaluated (Figure 8). The lesion remains filled with the implant. Further it is observed that the implant is colonised with cells originating from the underlying bone marrow. Cells were encountered in the settled thermosensitive chitosan hydrogel (B) as well as in the chitosan alginate beads (C) (chitosan trabeculae are indicated by D). This test confirms that the biphasic implant can

be easily handled and grafted. The biodegradable nature of the implant ensures a progressively resorption after implantation.

Example 8. Effect of chitosan molecular weight in beads formation.

Different molecular weights of native chitosan have been used in the process according to the invention. Different physical parameters such as pH and viscosity of the mixed solution (with chondrocytes) were measured., Osmolarity of the resulting hydrogel is measured according to a well-known technique in the art.

Table 1

Solutions	pH	Viscosity (Cps)	Osmolarity (mOsm/kg)
Alginate 1.2% / chitosan 22 kDa 0.6%	7.8	110.7	324
Alginate 1.2% / chitosan 30 kDa 0.6%	8.3	230	305
Alginate 1.2% / chitosan 32 kDa 0.6%	7.8	192	308
Alginate 1.2% / chitosan 55 kDa 0.6%	8.0	279	290
Alginate 1.2% / chitosan 55 kDa 0.6%	8.2	336	302
Alginate 1.2% / chitosan 91 kDa 0.6%	Impossible to mix		
Alginate 1.2% / chitosan 146 kDa 0.6%	Impossible to mix		

We concluded that mixed beads can be made with native chitosan below 55 kDa. For example, at 91 kDA, beads cannot be made using the process described in our invention and at the ratio alginate 1.2% and chitosan 0.6%, the chitosan solution is too viscous. Therefore, the selection of chitosan molecular weight is an essential element in our invention.

Example 9. Effect of chitosan molecular weight on chondrocytes behaviour

Molecular weight has an influence on the chondrocytes behaviour as illustrated in figures 3A, 5A and 6. The effect of chitosan 40 kDa on MMP-3, IL-6 and aggrecan is significantly different from that obtained with 20 kDa. Chitosan 20 kDa is less efficient on IL-6 and MMP-3 synthesis than 40 kDa chitosan, but more effective on aggrecan synthesis than 40 kDa. This clearly demonstrates that the biological activity of native chitosan is directly dependent of the molecular weight.

CLAIMS

1. A method of producing a hydrogel matrix comprising cartilage-forming cells said method comprising the steps of:
   - providing a solution of alginate,
   - providing a solution with chitosan of Mw lower than 60 kD,
   - mixing the solution of alginate and the solution of chitosan with cartilage-forming cells, wherein said mixed solution comprises between 0.5 and 0.7 % (w/v) of chitosan and between 1 and 1.4 % alginate (w/v),
   - introducing droplets of said mixed solution into a solution with Ca^{2+} or Sr^{2+} cations, and
   - isolating polymerised beads from said solution with cations.

2. The method according to claim 1, wherein said mixed solution comprises 0.6 % chitosan.

3. The method according to claim 1 or 2, wherein said mixed solution comprises 1.2 % alginate.

4. The method according to any one of claim 1 to 3, wherein alginate and chitosan in said mixed solution are present in a ratio between 1.75 and 2.25.

5. The method according to any one of claims 1 to 4, wherein said chitosan has a Mw of between 35 and 45 kD.

6. The method according to any one of claims 1 to 5, further comprising the step of cultivating said beads comprising cartilage-forming cells in a growth medium.

7. The method according to claim 6, wherein said growth medium comprises serum.

8. The method according to any one of claims 1 to 7, wherein said droplets are passed through a needle to obtain a particle with a diameter between 0.01 and 5 mm.

9. A spherical hydrogel bead with a diameter between 0.01 and 5 mm, comprising a homogeneous mixture of alginate and chitosan of Mw lower than 60 kD and further comprising cartilage-forming cells within said matrix, characterised in that said bead comprises between 1 and 1.4 % alginate and between 0.5 and 0.7 % chitosan.

10. The bead according to claim 9, wherein said bead comprises 1.2 % alginate.

11. The bead according to claim 9 or 10, wherein said bead comprises 0.6% chitosan.

12. The bead according to any one of claims 9 to 11, wherein the chitosan has a Mw between 35 and 45 kD.

13. The bead according to any one of claims 9 to 12, wherein the cartilage-forming cells, after 21 days of cultivation express below 0.02 units alkaline phosphatase per ng LDH, when grown in the presence of serum.

14. Use of cartilage-forming cells comprised in a bead according to any of claims 9 to 12, for the repair of cartilage defects.

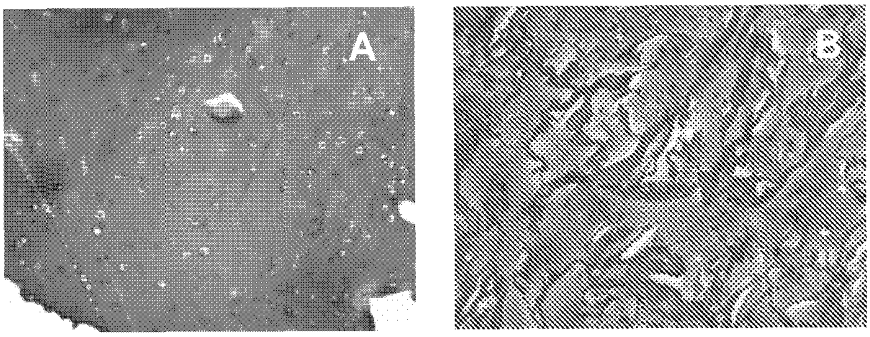

Figure 1

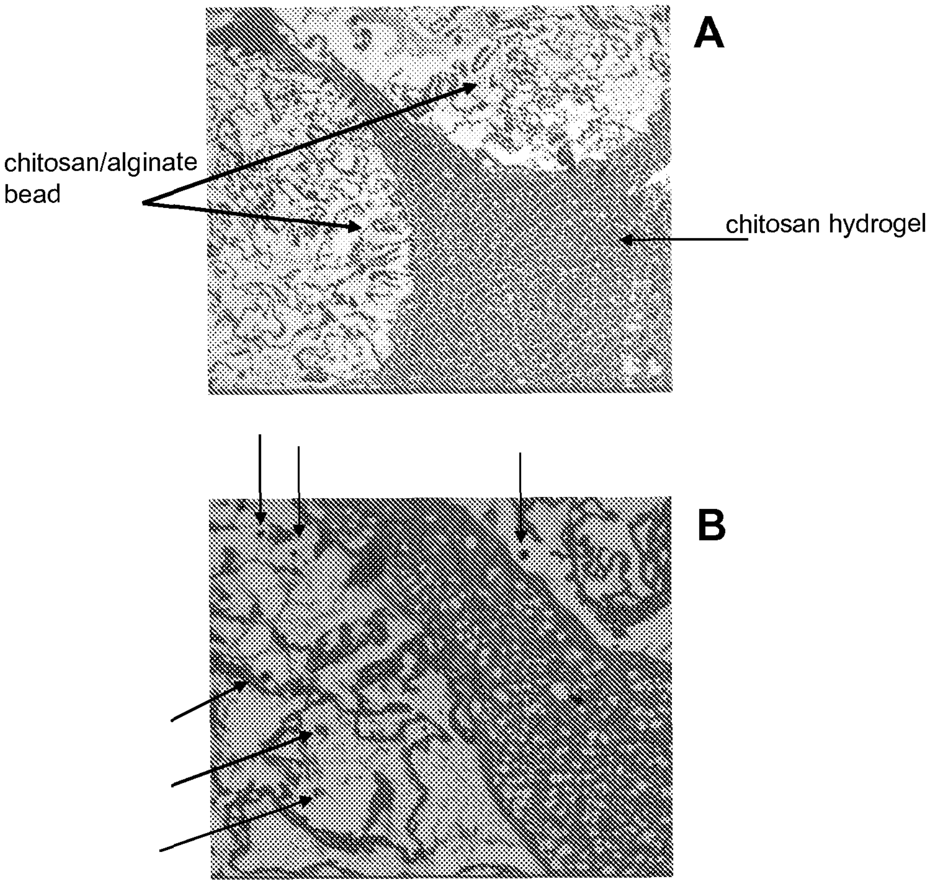

Figure 2

A

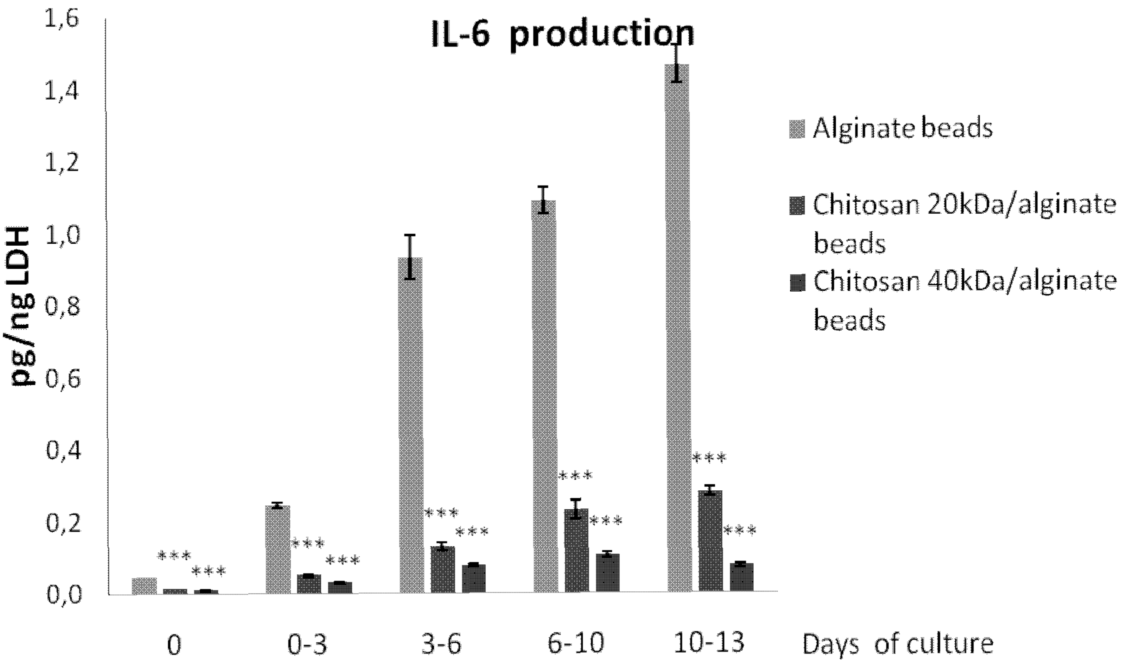
IL-6 production
1,6
1,4
1,2
1,0
0,8
0,6
0,4
0,2
0,0
pg/ng LDH
Alginate beads
Chitosan 20kDa/alginate beads
Chitosan 40kDa/alginate beads
***
0
0-3
3-6
6-10
10-13
Days of culture

B

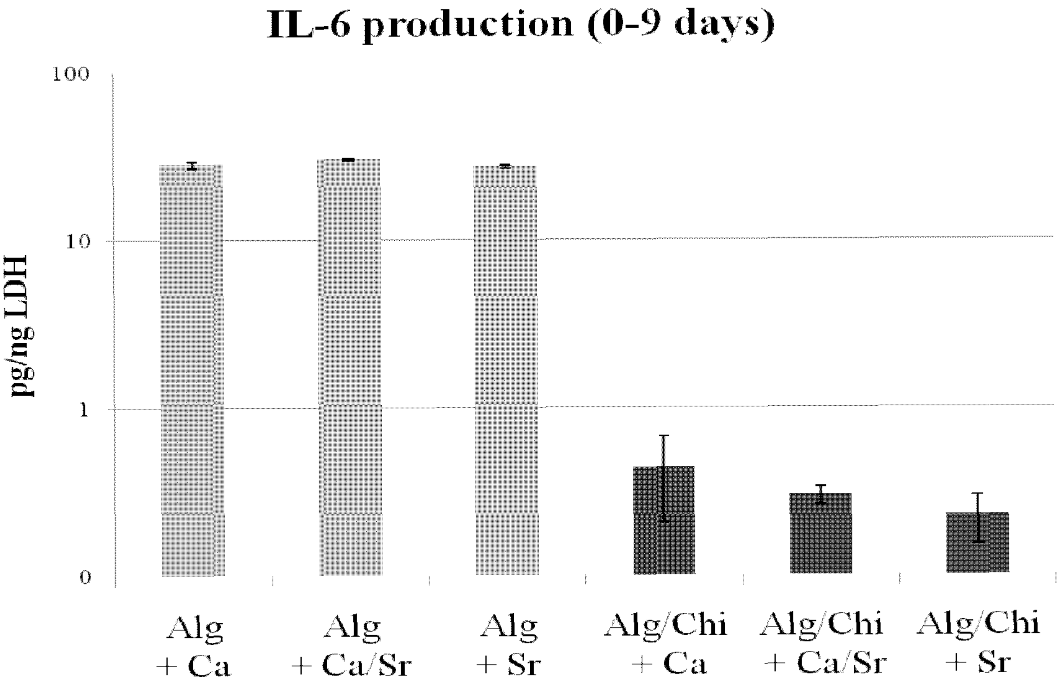
IL-6 production (0-9 days)
100
10
1
0
pg/ng LDH
Alg + Ca
Alg + Ca/Sr
Alg + Sr
Alg/Chi + Ca
Alg/Chi + Ca/Sr
Alg/Chi + Sr

Figure 3

A

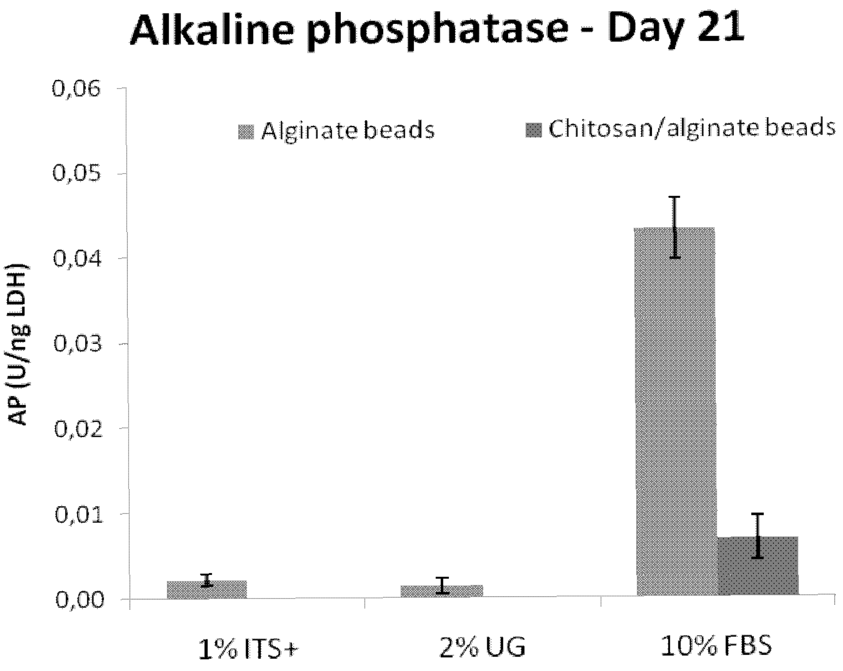
Alkaline phosphatase - Day 21
0,06
0,05
0,04
0,03
0,02
0,01
0,00
AP (U/ng LDH)
Alginate beads
Chitosan/alginate beads
1% ITS+
2% UG
10% FBS

B

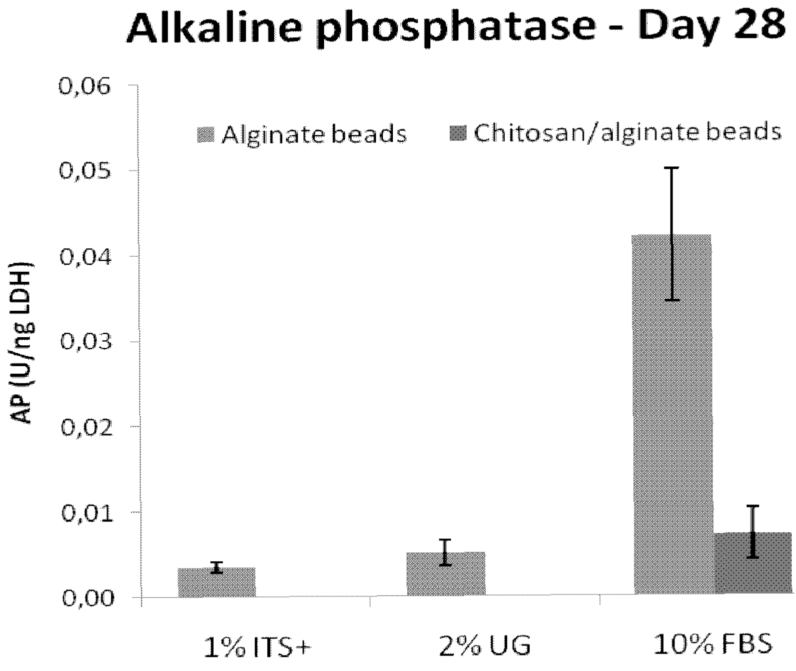
Alkaline phosphatase - Day 28
0,06
0,05
0,04
0,03
0,02
0,01
0,00
AP (U/ng LDH)
Alginate beads
Chitosan/alginate beads
1% ITS+
2% UG
10% FBS

Figure 4

A

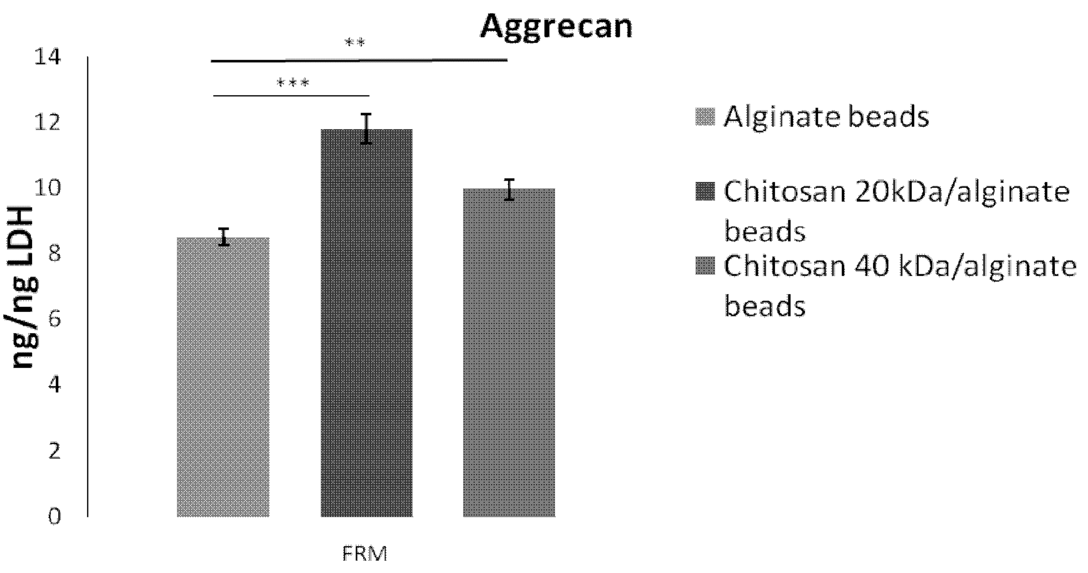
Aggrecan
**
***
14
12
10
8
6
4
2
0
ng/ng LDH
FRM
Alginate beads
Chitosan 20kDa/alginate beads
Chitosan 40 kDa/alginate beads

B

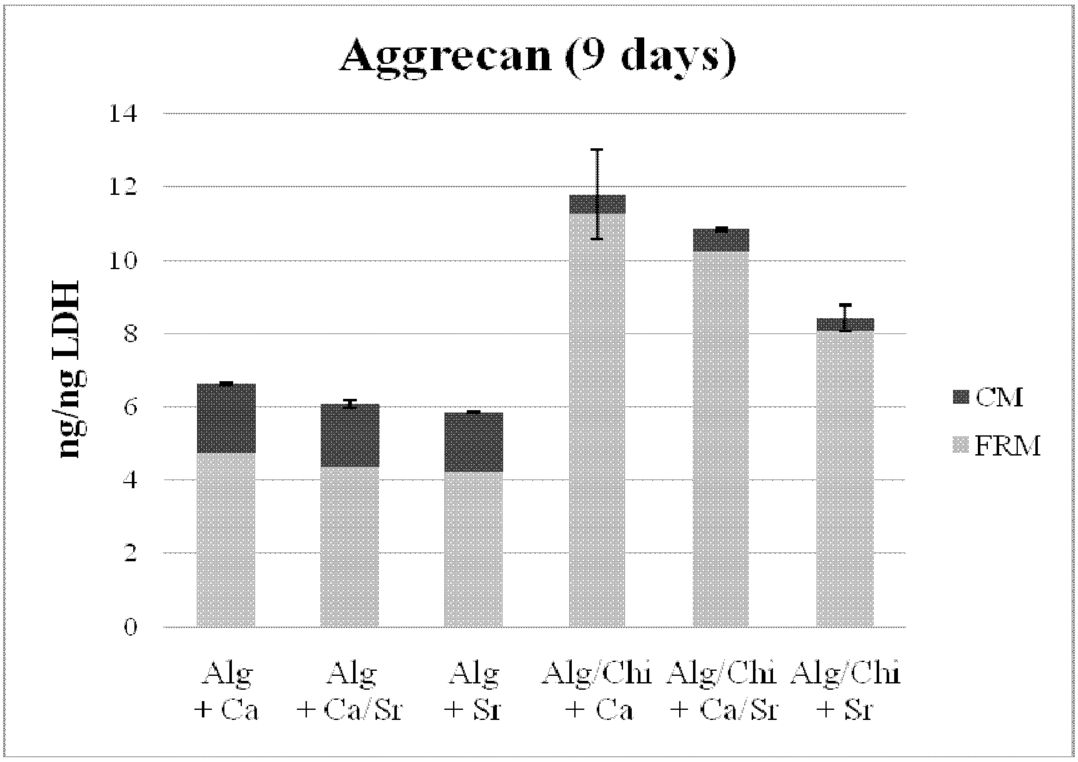
Aggrecan (9 days)
14
12
10
8
6
4
2
0
ng/ng LDH
Alg + Ca
Alg + Ca/Sr
Alg + Sr
Alg/Chi + Ca
Alg/Chi + Ca/Sr
Alg/Chi + Sr
CM
FRM

Figure 5

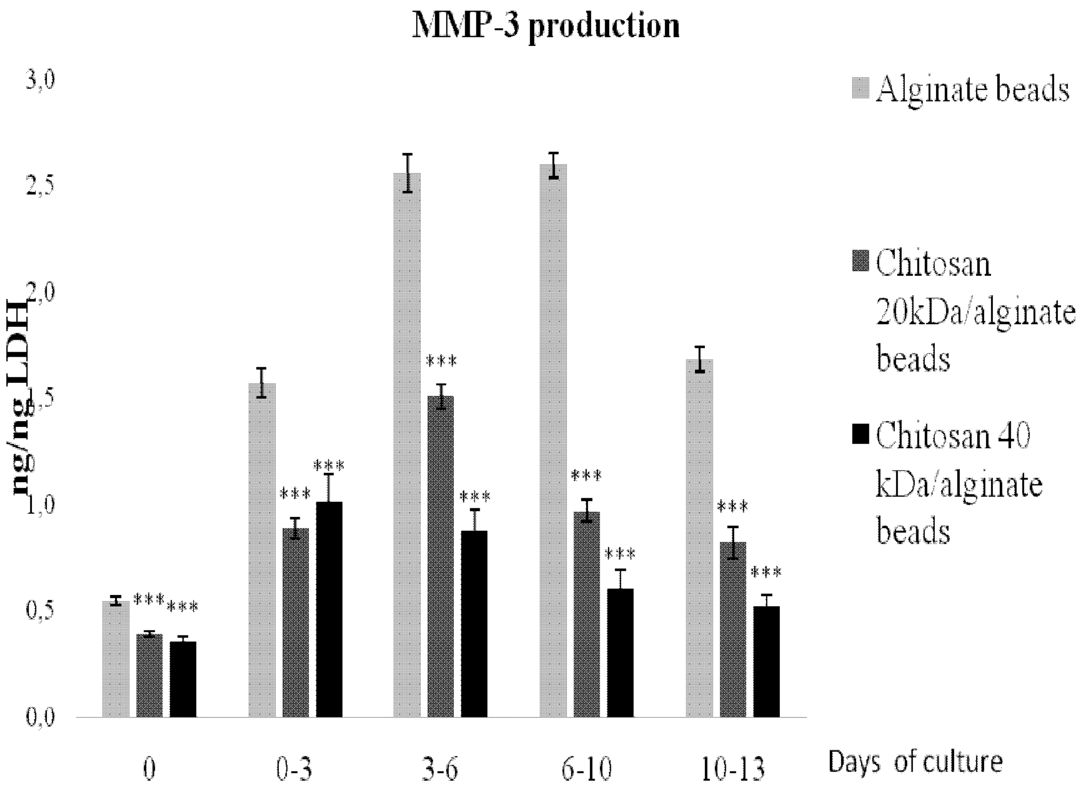
MMP-3 production
3,0
2,5
2,0
1,5
1,0
0,5
0,0
ng/ng LDH
***
Alginate beads
Chitosan 20kDa/alginate beads
Chitosan 40 kDa/alginate beads
0
0-3
3-6
6-10
10-13
Days of culture

Figure 6

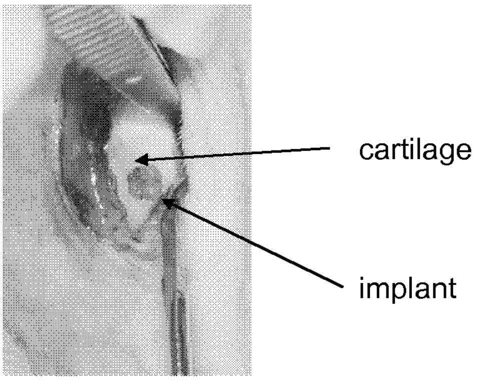

Figure 7

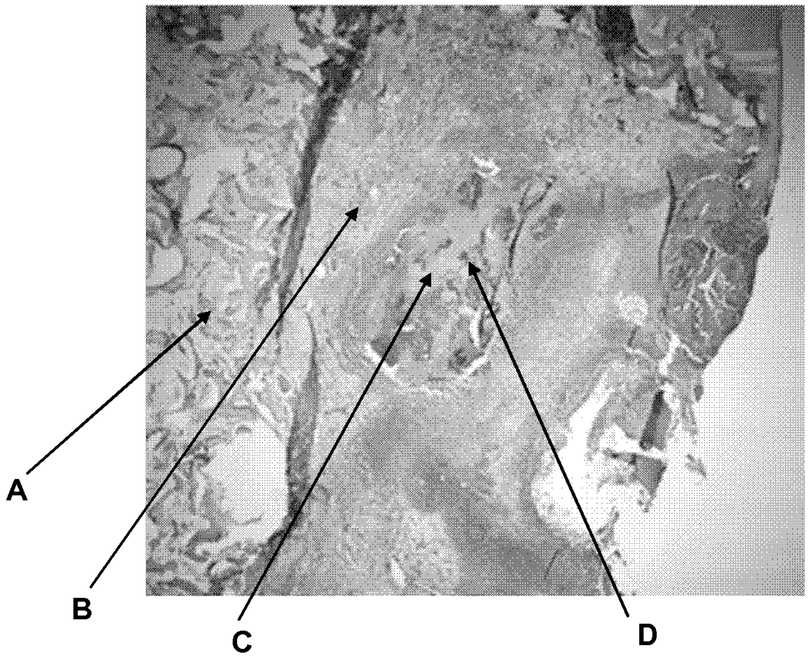

Figure 8

INTERNATIONAL SEARCH REPORT

International application No
PCT/EP2011/052013

A. CLASSIFICATION OF SUBJECT MATTER
INV. A61L27/20 A61L27/38
ADD.

According to International Patent Classification (IPC) or to both national classification and IPC

B. FIELDS SEARCHED

Minimum documentation searched (classification system followed by classification symbols)
A61L C12N

Documentation searched other than minimum documentation to the extent that such documents are included in the fields searched

Electronic data base consulted during the international search (name of data base and, where practical, search terms used)

EPO-Internal, WPI Data

C. DOCUMENTS CONSIDERED TO BE RELEVANT

Category*	Citation of document, with indication, where appropriate, of the relevant passages	Relevant to claim No.
A	WO 2007/135114 A1 (UNI DEGLI STUDI DI TRIESTE [IT]; PAOLETTI SERGIO [IT]; DONATI IVAN [IT) 29 November 2007 (2007-11-29)<br>page 13, line 8 - page 14, line 6<br>page 15, lines 4-27; example 12<br>page 25, lines 7-28<br>claims<br>-----<br>-/--	1-14

[X] Further documents are listed in the continuation of Box C.

[X] See patent family annex.

* Special categories of cited documents :

"A" document defining the general state of the art which is not considered to be of particular relevance

"E" earlier document but published on or after the international filing date

"L" document which may throw doubts on priority claim(s) or which is cited to establish the publication date of another citation or other special reason (as specified)

"O" document referring to an oral disclosure, use, exhibition or other means

"P" document published prior to the international filing date but later than the priority date claimed

"T" later document published after the international filing date or priority date and not in conflict with the application but cited to understand the principle or theory underlying the invention

"X" document of particular relevance; the claimed invention cannot be considered novel or cannot be considered to involve an inventive step when the document is taken alone

"Y" document of particular relevance; the claimed invention cannot be considered to involve an inventive step when the document is combined with one or more other such documents, such combination being obvious to a person skilled in the art.

"&" document member of the same patent family

Date of the actual completion of the international search	Date of mailing of the international search report
30 March 2011	05/04/2011

Name and mailing address of the ISA/	Authorized officer
European Patent Office, P.B. 5818 Patentlaan 2<br>NL - 2280 HV Rijswijk<br>Tel. (+31-70) 340-2040,<br>Fax: (+31-70) 340-3016	Van den Bulcke, H

Form PCT/ISA/210 (second sheet) (April 2005)

C(Continuation). DOCUMENTS CONSIDERED TO BE RELEVANT

Category*	Citation of document, with indication, where appropriate, of the relevant passages	Relevant to claim No.
A	SEDA TIGLI R ET AL: "Evaluation of alginate-chitosan semi IPNs as cartilage scaffolds", JOURNAL OF MATERIALS SCIENCE: MATERIALS IN MEDICINE, KLUWER ACADEMIC PUBLISHERS, BO, vol. 20, no. 3, 6 November 2008 (2008-11-06), pages 699-709, XP019680168, ISSN: 1573-4838 paragraphs [02.3], [2.5], [2.5.1], [3.4.1] -----	1-14
A	US 2004/047912 A1 (BARDONNET RAPHAEL [FR] ET AL) 11 March 2004 (2004-03-11) page 1, paragraph 23 - page 2, paragraph 57 claims -----	1-14
A	BARUCH, L.; MACHLUF, M.: "Alginate-Chitosan Complex Coacervation for Cell Encapsulation: Effect on Mechanical Properties and on Long-Term Viability", BIOPOLYMERS, vol. 82, 21 March 2006 (2006-03-21), pages 570-579, XP002593055, the whole document -----	1-14

INTERNATIONAL SEARCH REPORT

Information on patent family members

International application No
PCT/EP2011/052013

Patent document cited in search report	Publication date	Patent family member(s)	Publication date
WO 2007135114 A1	29-11-2007	AT 454405 T	15-01-2010
		CA 2652967 A1	29-11-2007
		CN 101454348 A	10-06-2009
		EP 2029629 A1	04-03-2009
		JP 2009537268 T	29-10-2009
		US 2009202640 A1	13-08-2009
US 2004047912 A1	11-03-2004	AT 427340 T	15-04-2009
		EP 1355981 A1	29-10-2003
		ES 2325313 T3	01-09-2009
		FR 2819814 A1	26-07-2002
		WO 02057355 A1	25-07-2002
